# Washing scaling of GeneChip microarray expression

**DOI:** 10.1186/1471-2105-11-291

**Published:** 2010-05-28

**Authors:** Hans Binder, Knut Krohn, Conrad J Burden

**Affiliations:** 1 Interdisciplinary Centre for Bioinformatics; Universität Leipzig, D-4107 Leipzig, Haertelstr. 16-18, Germany; 2 LIFE Center; Universität Leipzig, D-4103 Leipzig, Philipp-Rosenthalstr. 27, Germany; 3 Interdisciplinary Center for Clinical Research, Medical Faculty; Universität Leipzig, D-04107 Leipzig, Inselstr. 22, Germany; 4 Mathematical Sciences Institute, Australian National University, Canberra, A.C.T.0200, Australia

## Abstract

**Background:**

Post-hybridization washing is an essential part of microarray experiments. Both the quality of the experimental washing protocol and adequate consideration of washing in intensity calibration ultimately affect the quality of the expression estimates extracted from the microarray intensities.

**Results:**

We conducted experiments on GeneChip microarrays with altered protocols for washing, scanning and staining to study the probe-level intensity changes as a function of the number of washing cycles. For calibration and analysis of the intensity data we make use of the 'hook' method which allows intensity contributions due to non-specific and specific hybridization of perfect match (PM) and mismatch (MM) probes to be disentangled in a sequence specific manner. On average, washing according to the standard protocol removes about 90% of the non-specific background and about 30-50% and less than 10% of the specific targets from the MM and PM, respectively. Analysis of the washing kinetics shows that the signal-to-noise ratio doubles roughly every ten stringent washing cycles. Washing can be characterized by time-dependent rate constants which reflect the heterogeneous character of target binding to microarray probes. We propose an empirical washing function which estimates the survival of probe bound targets. It depends on the intensity contribution due to specific and non-specific hybridization per probe which can be estimated for each probe using existing methods. The washing function allows probe intensities to be calibrated for the effect of washing. On a relative scale, proper calibration for washing markedly increases expression measures, especially in the limit of small and large values.

**Conclusions:**

Washing is among the factors which potentially distort expression measures. The proposed first-order correction method allows direct implementation in existing calibration algorithms for microarray data. We provide an experimental 'washing data set' which might be used by the community for developing amendments of the washing correction.

## Background

Gene expression profiling using microarrays has become a standard technique for the large scale estimation of transcript abundance [1]. The method is based on the hybridization of RNA prepared from samples of interest with gene-specific oligonucleotides attached to the array surface. Following hybridization, the experimental protocol comprises the labeling of the bound RNA targets with fluorescent markers, a post-hybridization washing procedure (fluidic script) and the optical detection of probe-bound targets 'surviving' the washes. The washing step aims at improving the signal-to-noise ratio by removing free optical markers and hybridized nonspecific targets with the purpose of increasing the relative contribution of the specific signal. The scanned intensity of the probe spots of each array are subsequently calibrated to obtain exact estimates of the expression levels of tens of thousands of genes in one measurement.

Both the quality of washing achieved in the experimental protocol and the adequate consideration of the washing mechanism in intensity calibration ultimately affect the quality of the expression estimates extracted from the microarray intensities and subsequent downstream analysis. Post-hybridization washing is an essential part of any microarray experiment irrespective of the technology used (two-color or single intensity detection, RNA or DNA target hybridization, high- or low-density probe spots, long- or short-sequence oligomers) or of the intended application (expression profiling, genotyping and copy number measurements, micro-RNA detection and/or re-sequencing tasks).

Previous experimental studies have been conducted to optimize the hybridization and washing conditions and/or to discover basic mechanisms of washing as function of probe/target duplex stability and of the applied washing protocol [2-5]. Furthermore, theoretical approaches have been published which explicitly take into account washing and introduced 'washing terms' to improve the agreement with experimental spiked-in data [6-9]. Spike-in experiments typically provide the intensities of a set of selected probes as a function of their target concentration. It was found that perfect match (PM) and single-mismatched (MM) probes show different asymptotic intensity levels at saturation conditions. This result contradicts equilibrium thermodynamics of surface adsorption predicting equal saturation levels for all probes independent of their sequence specific binding affinity. Burden et al. [9] showed that post hybridization washing explains the observed discrepancy: Sequence-dependent dissociation of the probes in the absence of free targets decreases the amount of probe-bound targets differently for PM and MM probes, resulting in observed intensity differences. This hypothesis was later confirmed by the 'washing' experiments of Skvortsov et al [4] on Affymetrix GeneChip arrays. The authors applied customized fluidic scripts and selective labeling of specific and non-specific targets and measured the respective signal components prior to and after stringent washes to estimate the washing yield in dependence of the hybridization mode, target concentration and equilibration time prior to washing.

Our study continues these previous approaches in experimental and theoretical respects to get further insights into the detailed probe-level kinetics of washing and the underlying mechanism. We estimate the systematic error of the expression estimates obtained from calibration methods not considering washes and develop appropriate corrections. To our best knowledge there is no study so far which estimates the effect of washing on the expression estimates and there is no calibration algorithm for appropriate correction.

In particular, we conducted microarray experiments with altered protocols for washing, scanning and staining to study the resulting intensity changes as a function of washing cycles. A second staining round was applied to estimate the effect of labeling on the washing efficiency. For calibration and analysis of the intensity data we make use of the 'hook' method [10,11] which disentangles intensity contributions due to non-specific and specific hybridization without special labeling or spike-in experiments. The method provides a set of well-defined parameters characterizing the state of hybridization and its change upon washing. The method also enables information about the sequence specificity of washing to be extracted. Based on the results of our analyses we will propose modifications of the hook method to correct probe intensities for the effect of washing and thus improve the accuracy of expression estimates.

The paper is laid out as follows. Section 2 sets out the microarray experiments and the theoretical basics of the intensity response of microarray probes including the post-hybridization washing step. In Section 3 different aspects of the washing data are analyzed: the hybridization mode of the probes, the probe-type (PM or MM probes), the relation between intensity and the washing yield, the washing kinetics in the limit of a small and a large number of washing cycles, the sequence dependence of washing and the effect of labeling. The discussion in Section 4 addresses issues such as the 'washing scaling' of expression estimates in terms of the systematic bias of intensity calibration methods due to washing. Finally we propose a practicable correction for the systematic effect of washing. The theoretical approaches and models used are described in detail in the theoretical part given at the end of the paper. It includes the extension of the hook method to post-hybridization washing which is applied for data analysis. In the supplementary material we give a further example by re-analyzing parts of the washing experiment of Skvortzov et al. [4] using the hook method.

## Results

### Washing experiments

Aliquots of the RNA sample solution were hybridized to three Affymetrix GeneChip HGU133plus2 arrays (A to C) and equilibrated for 16 hours in the hybridization oven. For one of the three arrays (C) washing and staining was performed according to the manufacturer's instructions. Briefly, the standard protocol includes a low stringent wash (900 mM Na^+^) at 30°C followed by 6 stringent wash cycles at 50°C with decreased salt concentration (100 mM Na^+^). After these washes the array is stained with streptavidin phycoerythrin (SAPE) in two rounds which are intermitted by a round of anti-SAPE antibody staining (staining step) and non-stringent washes.

**Figure 1:**
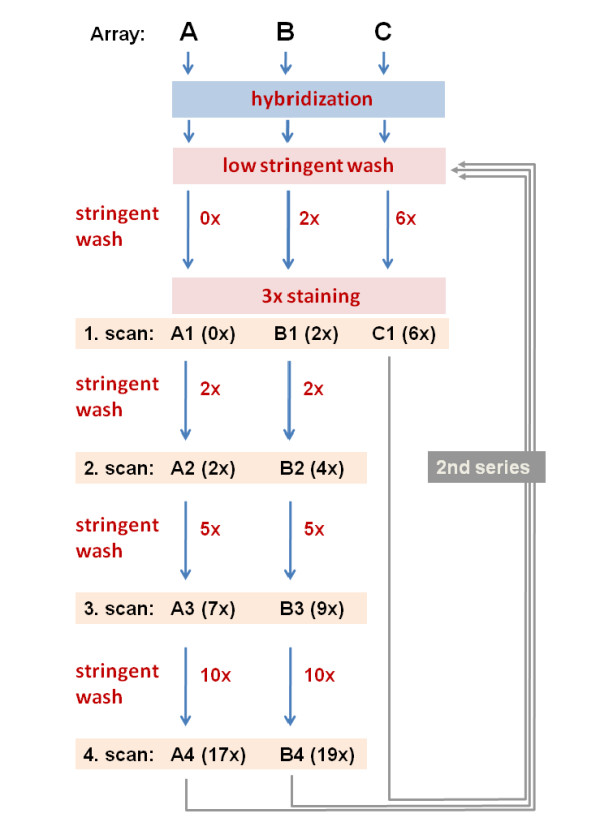
**Workflow of the washing experiment**: Three human genome arrays (HG-U133plus2) were hybridized with identical RNA-samples, equilibrated for 16 hours, low stringently washed and stained (labeled) using the same protocol. Stringent wash cycles and subsequent scans were applied using different protocols: Two arrays, A and B, were stringently washed and scanned in four alternating cycles where in each cycle the washing step was repeated several times as indicated. Chip C was processed using the standard protocol of six stringent washes before staining. The chip measurements are assigned according to 'chip-cycle number-(total number of washings)', e.g. A-3(7x). After finishing the first series of wash/scan-cycles the whole procedure was repeated a second time.

For another array (A) the first scan was done immediately after low stringent wash and staining without subsequent stringent washing. Then the array was stringently washed and scanned in alternating order three more times where each washing step consists of a definite number of washing cycles (see Figure 1). The third array (B) was low stringently washed followed by two stringent washing cycles and staining before the first scan. Subsequently it was analogously processed as array A. Each measurement (scan) is characterized by the array (A, B or C), the scan number and the total number of washing cycles performed after hybridization. The first and second scans of array A (A1 and A2) resemble the design of the previous washing experiment [4].

All three chips are repeatedly processed in a second series of alternating wash/scan-cycles which was performed using the same protocol for each chip as in the first series as described above. As in the first series the arrays were also stained a second time to compensate for any loss of bleached fluorescent dye. The set of raw intensity data (cel-files) is available from the Gene Expression Omnibus (GEO) repository under accession number GSE18161.

Selected results of a pre-experiment performed to test the fluidic script of the washing-scanning cycles are given in the additional file 1 (supplementary text) to support the results of the main experiment described in the remainder of the paper. The results of this pre-experiment also served as basis for the design of the main experiment as discussed in the supplement.

### Hybridization and washing of microarrays: Theory and basic equations

Microarray experiments include several steps: (i) RNA-extraction, purification and preparation which includes amplification, in vitro transcription and biotin-labelling; (ii) hybridization, i.e. the addition of the RNA-sample onto the microarray and equilibration during which the added RNA-fragments are intended to bind to the oligonucleotide probes attached to the chip surface; (iii) staining, i.e. the addition of fluorescent marker (streptavidin-phycoerythrin; SAPE) which bind to the biotin-labels covalently attached to a certain fraction of cytosines of the hybridized RNA-fragments. Primary SAPE association is further amplified in a second round of SAPE-to-SAPE binding via anti-SAPE antibody staining; (iv) washing, i.e. rinsing of the chip with buffer. Essentially two washing regimes are applied, namely a mild low-stringent one intended to remove predominantly unhybridized markers and RNA-targets; and a more severe high stringent regime to wash off weakly bound non-specific RNA-fragments; (v) scanning and subsequent image analysis to quantify the probe intensities.

The hybridization step (ii) can be described by two coupled reversible second-order reactions of specific (S) and non-specific (N) target binding to the microarray probes,(1)

The superscript "f" indicates free species and PS and PN are the probes duplexed with specific and non-specific transcripts, respectively. K^P,S ^and K^P,N ^are the equilibrium constants of specific and non-specific transcript binding. The superscript 'P' accounts for the fact that the constants depend on the particular sequence of each probe and thus they vary in a probe-specific fashion. In particular we will use P = PM,MM below to differentiate between the properties of perfect match (PM) and mismatched (MM) probes used on GeneChip microarrays.

The reactions (1) provide the hyperbolic adsorption law for the probe/target duplex formation under equilibrium conditions,(2)

where Θ^P,h^(0) is the fraction of probes occupied by species 'h' immediately after the hybridization step. The square brackets denote the concentrations of the respective species. The so-called binding strengths are defined as(3)

[h] = [N], [S] are the total concentrations of the respective transcripts.

Upon washing the microarray is rinsed with RNA-free buffer solution which causes the partial unbinding of specific and non-specific transcripts according to [6,8,9](4)

where the k^P,h ^denote the dissociation rate constants upon washing. Eq. (4) assumes that the supernatant solution acts as a concentration sink which removes the unbound transcripts from the system. This assumption seems reliable because in practice washing is performed in discrete cycles in each of which the exhausted buffer is replaced by new one (see also the detailed discussion of the process given in [6]). This first order reaction kinetics gives rise to the exponentially decaying *washing function *[3,12],(5)

which provides the fraction of probe/target duplexes surviving after washing time t. If each washing cycle is performed using the same protocol (amount of buffer and dwell-time of the buffer in the cell) then the argument of the washing function can be substituted by the number of washing cycles. In this case w^P,h^(t) with t = 1, 2,... defines the reduction of the probe occupancy after t washing cycles.

We assume in agreement with previous studies [8,9] that the dissociation rate is related to the stability of the respective duplex in terms its free energy of probe/target hybridization, which in turn depends on the equilibrium binding constant introduced in Eq. (1), ΔG^P,h ^= -RT·lnK^P,h ^(R is the gas constant), i.e.(6)

where const = K_0_^γ ^and γ are sequence-independent scaling constants which apply to all probes on the microarray. K_0_, for example, depends on the rate constant of probe/target formation and the washing conditions (salt concentrations, temperature, see [6] for a detailed argumentation and references given therein).

The probe intensities measured in the scanning step (v) are directly related to the total probe occupancy due to specific and non-specific binding surviving after t washing cycles, [9,13-16] (see Eqs. (2) and (5)),(7)

where M(t) is the maximum intensity upon complete saturation of the probes and O(t) its minimum value due to the optical background. The maximum intensity rescales the dimensionless occupancy into intensity units. It depends on the amount and quality of optical labelling of the targets, on the sensitivity of the scanner and on the imaging software which transforms the intensity spot of each probe into one intensity value. Saturation of optical detection is typically characterized by the appearance of a constant upper limit of intensity values which was not observed in most of our data. We assume therefore linear and time-independent calibration of the scanner. Exceptions observed in the 2^nd ^series will be discussed below. More critically, repeated scanning will potentially bleach the fluorescent labels [17,18]. Such bleaching gives rise to a time-dependence of M(t)≈ M·b(t), where b(t) is the bleaching factor decaying with time, i.e. b(0) = 1 and 1 ≥ b(t > 0) ≥ 0. The optical background depends, besides other factors, on the amount of residual fluorescent markers and thus is also basically a function of washing and scanning cycles due to washing and bleaching as well. Throughout the paper we will consider net intensities which have been corrected for the optical background before further analysis, I^p ^(t) = I^p ^* (t) -O(t), using, the zone-algorithm provided by Affymetrix for estimating O(t) for each chip measurement [19].

### Washing efficiency is related to intensity

**Figure 2:**
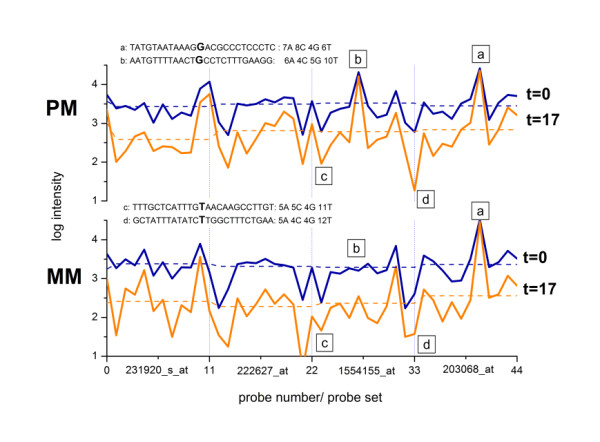
**PM- and MM-probe intensities of four selected probe sets before (t = 0) and after (t = 17) washing**: Each probe set contains eleven PM and MM probes. Washing affects the different probes in a selective fashion. For example, the high-intensity PM-probes a and b (see labels in the figure) remain nearly unaffected, wheras the weak-intensity probes c and d respond strongly to washing. The horizontal dashed lines are the mean intensities which are log-averaged over all eleven probes of each probe set. The figure shows probes with relatively large set-averaged intensities which are predominantly hybridized with specific transcripts. The sequences of the four labeled probes (a-d) are explicitly given together with the total number of adenines, cytosines, guanines and thymines per sequence. Note that there are no obvious correlations between the given sequences and the intensity changes owing to washing.

Figure 2 compares the intensities of the PM- and MM-probe intensities of four selected probe sets before (t = 0) and after t = 17 stringent washing cycles taken from scans A1 and A4 (see Figure 1). The mean intensity level clearly decreases after washing. The mean decrement of the MM slightly exceeds that of the PM probes (see the horizontal dashed lines in Figure 2).

Each probe set is intended to interrogate one transcript. The target concentration is therefore assumed to be a constant for all probes of each set in a first order approximation which neglects effects such as the 3'/5'-amplification bias of RNA-fragments. The observed variability of the intensities of the individual probes about their set-average results mainly from the sequence dependence of the binding constant for probe/target association. In Figure 2 two probes of relatively high (see labels a and b) and two probes of relatively low (labels c and d) intensity are indicated. Washing leaves the intensities of the former ones nearly unchanged whereas the intensities of the latter probes strongly decrease. Both PM and MM behave similarly. The scattering width of the probes about their set average clearly increases after washing.

**Figure 3:**
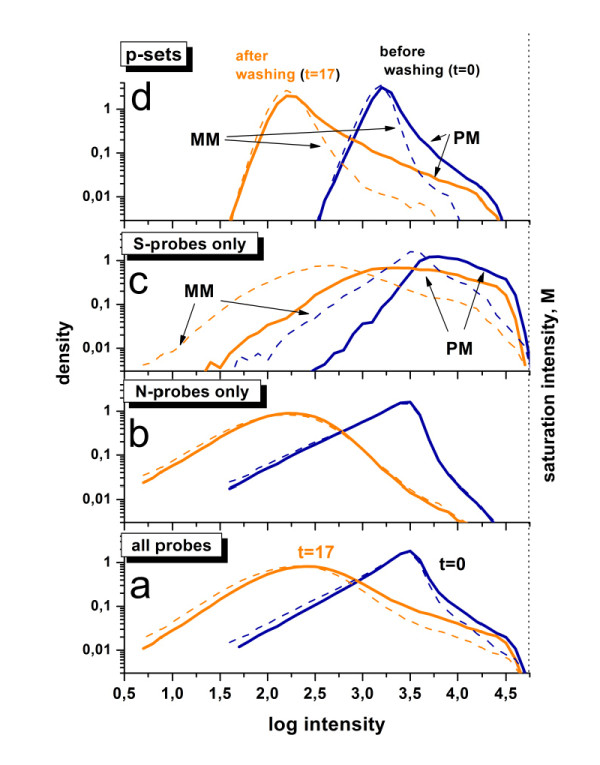
**Probe intensity distributions before (t = 0) and after (t = 17 cycles) washing**: Thick and thin lines refer to PM- and MM-probes of chip A, respectively. The different panels show the distributions of: (a) all probes of the array (~6·10^5 ^probes), (b) probes predominantly hybridized with non-specific transcripts (2·10^5 ^probes) and (c) probes predominantly hybridized with specific transcripts (2·10^4 ^probes). Accordingly, about 4·10^5 ^probes are not considered because they are significantly hybridized by non-specific and specific transcripts as well. Panel d shows the respective distributions of set-averaged log-intensities. The vertical dotted line at the right indicates the maximum intensity M referring to complete saturation of the probe spots.

To generalize these trends we calculate the intensity distributions of all PM- and MM-probes of the respective chip measured in the first and the last scan after t = 0 and t = 17 washing cycles (see Figure 3 and also Figure 1 for experimental protocol). Basically, washing broadens the intensity distribution and shifts their center to the left. The high-intensity limit of the right flank of the distribution remains essentially unaffected whereas the low intensity flank considerably shifts towards smaller intensities (panel a of Figure 3). Washing obviously affects the probes in an intensity-dependent manner. The decrease of the effect of washing with increasing intensity can be explained by the fact that higher intensities are associated with stronger probe/target interactions which in turn are relatively stable against washing.

The probe intensity decomposes additively into contributions due to non-specific and specific hybridization (see Eq. (7)). To study the effect of washing on both hybridization modes we separately calculate the intensity distributions for probes which are predominantly hybridized with non-specific (panel b of Figure 3) and with specific transcripts (panel c). The respective ensembles of probes are obtained using the classification criteria provided by the hook method as described below.

Washing has an almost identical effect on the non-specifically hybridized PM and MM probes, their respective distributions being almost identical both before and after washing. This observation can be explained by the fact that the discrimination between the probe-types PM versus MM is relevant only with respect to the specific transcripts not to non-specific transcripts.

On the other hand, PM and MM probes respond differently under specific hybridization: the distributions of the MM probes before and after washing are shifted towards smaller intensities compared with the respective PM-distributions. The smaller on-average intensity of the MM signal reflects the weaker binding of specific transcripts to these probes, owing to the mismatched pairing at the middle position of their probe sequence. Note that the high-intensity flank of the specifically hybridized probes remains essentially fixed after washing whereas the left flank shifts downwards considerably. The steep decay of the right, washing-independent flank of the PM-density distribution can be attributed to the maximum intensity value referring to saturated probe spots with strongly bound specific transcripts (see the vertical dotted line in Figure 3).

Note that bleaching is independent of the hybridization mode and thus it is expected to affect all probe intensities equally. Its effect on the time dependence of the intensity will therefore not exceed the weakest time course observed in repeated scanning. The virtual invariance of the specifically hybridized probes of largest intensity (see Figure 3c and probes a and b in Figure 2) gives consequently strong indication that bleaching adds, if at all, only a tiny contribution to the time dependence of the intensities provided that the scanner works below the saturation level of its calibration curve [20]. Saturation of the scanner is observed for a small fraction of probes in the 2^nd ^series but not in the 1^st ^one (see below). We will therefore neglect bleaching in the remainder of the paper to a good approximation.

Part d of Figure 3 shows the distributions of the log-averaged intensities for each probe set probing the same transcript. Trivially, averaging considerably reduces the variability of the intensity values giving rise to the narrowing of the distributions. Moreover, averaging over the probe set partly removes the sequence dependence of the intensities and this way stresses the effect of transcript abundance on the intensities. The respective distributions consequently reflect the effect of the varying amount of specific transcripts on the washing efficiency: Their right flank is governed essentially by specific hybridization (compare with panel c) whereas their peak and the left flank are obviously dominated by non-specific binding (compare with panel b). Note also that set-averaging reduces the apparent value of the saturation intensity because non-saturated spots also contribute to the average value.

### Probe-level kinetics of washing

**Figure 4:**
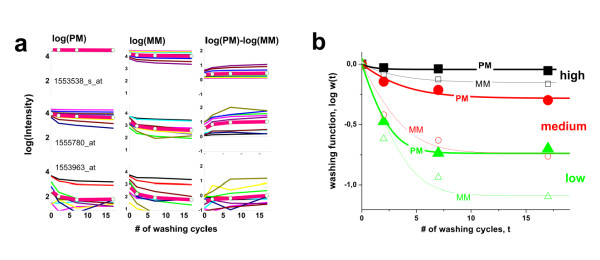
**Probe intensities as a function of the number of washing cycles**: Part a: The probes of three probe sets were selected for large (top row), intermediate (middle row) and low (bottom row) intensity levels (array A; see also Figure 1). The thick lines are the log-mean values averaged over the eleven probes of each probe set. The intensities of PM- and MM-probes and of their log-difference are shown from left to the right as indicated in the figure. Part b re-plots the averaged intensity data of the PM and MM shown in part a after normalization assuming a common start value of w(0) = 1 (symbols). The curves are calculated using Eq. (8).

Figure 4 (panel a) illustrates the effect of washing on the intensities of single P = PM and MM probes taken from three representative probe sets of high, medium and small average intensities. The obtained probe intensities decrease with increasing number of washing cycles, t, as expected. Moreover, the effect of washing decreases with intensity in agreement with the general trends discussed in the previous section. The decay law is obviously not single-exponential (Eq. (5)). Instead, it seems to follow a multiphase decay. This behavior can be rationalized in terms of heterogeneous desorption of different transcripts with different binding free energies. Let us approximate this behaviour using an empirical simple two-component decay function which considers a fast short-time and an asymptotic long-time component,(8)

Accordingly, the decay is characterized by two parameters: the exponential decay time τ^P ^(in units of the number of cycles after which the probe intensity is expected to decay to 1/e of its initial value) and the asymptotic intensity level, w^P^∞. The latter term neglects the kinetics of the slow component and subsumes its effect in terms of the fraction of probes which survived after extensive washing in the time-window of the experiment. Below we will apply a complementary approach to analyze the kinetics of the slow component more in detail.

Part b of Figure 4 shows examples of fits of Eq. (8) to the averaged PM and MM decays taken from part a of the figure (note the logarithmic scale). One finds that the limiting intensity values, log(w∞^P^), correlate with the initial intensities, logI(0), i.e. large intensity levels give rise to relatively large limiting values and vice versa. This correlation also becomes evident in the right column of Figure 4a which shows the log-difference of the PM- and MM-intensities of the considered probe pairs: The smaller MM-intensities of specifically hybridized probes are associated with faster decay rates compared with the respective PM-data, causing an increasing course of the log-difference in most cases.

To generalize these results we estimate the long- and short-time parameters for all PM and MM probes of the array A by means of(9)

**Figure 5:**
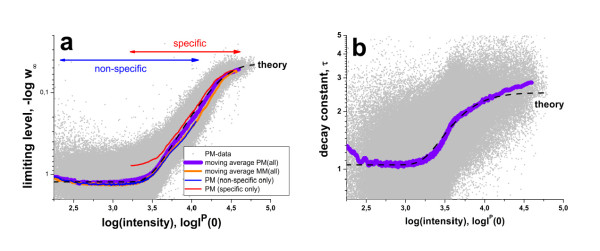
**Limiting values (panel a) and decay times (panel b) of the washing function as a function of the initial probe intensity logI^P^(0)**: The dots are the probe-level data of all PM-probes of array A (see Eqs. (8) and (9)). The moving average was calculated over 1000 probe-level data to extract the mean effect of intensity on both parameters (thick curves). The moving average of the MM probes (probe-level data are omitted for clarity) is virtually indistinguishable from that of the PM probes. The PM-data are also split into probes which are hybridized predominantly non-specifically and specifically (thin lines, see text). The respective moving averages cover the low-intensity and high intensity ranges, respectively, with considerable overlap (see arrows in panel a). These results show that the washing parameters are mainly determined by the probe intensities and thus by the binding constant independently of the probe type (PM or MM) and of the hybridization mode (specific or non-specific). The mean trends of w∞ and τ are well described using Eqs. (18) and (17) given in the Methods-section (dashed lines). Accordingly, the stepwise change of the washing parameters is governed by their power law dependence on the binding constant. The fits use a critical exponent of γ = 1.6 and the critical intensities of logI(0)^crit ^= 3.8 (for w∞) and 3.5 (for τ). The critical exponent and the critical intensity determine the sharpness of the sigmoidal change and the position of its inflection point, respectively (see also Figure 16 for illustration).

The obtained limiting values of the individual probes (see grey dots in panel a of Figure 5) are smoothed using a moving average over 10^3 ^probes to filter out the average relation between w∞^P ^and the initial intensity value, I^P^(0) (thick lines). It turns out that extensive washing reduces the intensities to about 10% of their initial values in a wide range of relatively small intensities, logI^P^(0) < 3.5. For intensities larger than a certain threshold, logI^P^(0) > 3.5, the limiting washing level increases with intensity up to w∞^P ^> 0.9 (i.e. -log w∞^P ^< 0.05 in Figure 5). In other words, up to 90% of the initial intensity value of probes of high intensity survives, whereas weak intensity probes are dimmed to less than 10% after extensive washing. Importantly, there is virtually no difference in the washing efficiency between the PM and MM probes indicating that both probe-types behave identically at the same intensity level. This result is consistent with the model of ref. [8].

The same result was obtained if one separately studies probes which predominantly hybridize with non-specific or specific transcripts (see thin lines in Figure 5) despite the fact that the respective probes accumulate in the low and high intensity range, respectively. These results show that the limiting washing level is governed by the probe intensity, independent of the probe type (PM or MM) and of the hybridization mode (specific or non-specific).

The decay constant shows a similar mean trend with increasing intensity as the limiting washing level despite the wider scattering of the individual probe data (panel b, Figure 5): Larger intensities are obviously associated with larger decay times τ^P ^indicating the slowing down of washing efficiency. Also the behaviour of this parameter is mainly driven by the intensity independently of probe type and hybridization mode (data not shown).

In the Methods-section we present a simple theoretical approach to express the two washing parameters studied as a function of the probe intensity. The theoretical curves obtained reproduce well the experimental data, and particularly the gradual increase of w∞ and τ at intensities above a certain threshold (see dashed curves in Figure 5). The theory assumes that the intensity is directly related to the probe/target-binding constant which in turn determines the washing rate in terms of a power law in agreement with Eq. (6). It gives rise to a relatively sharp intensity threshold above which both washing constants start to increase, in agreement with the experimental data.

### Selective washing of PM and MM probes and apparent concentration dependence

**Figure 6:**
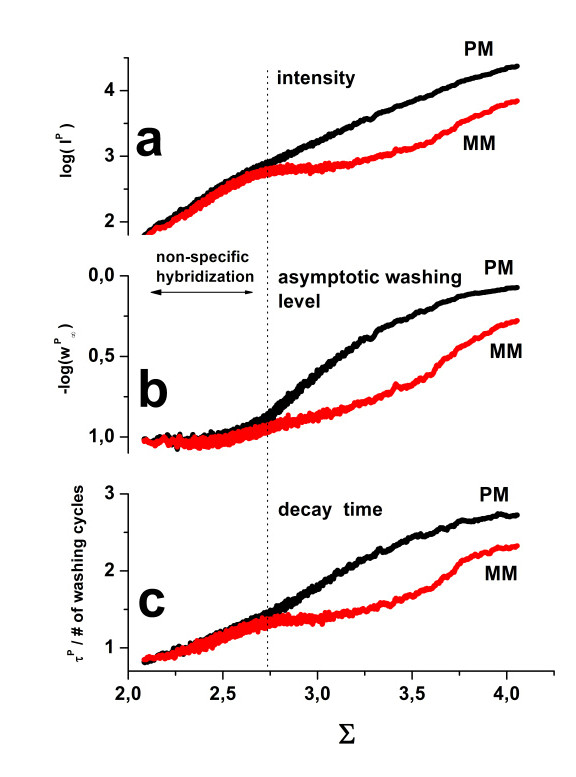
**PM- and MM-characteristics of washing**: Mean probe intensity (panel a), the asymptotic washing level (panel b) and the initial washing decay time (panel c) are shown as a function of the set-averaged probe intensity, Σ, which roughly estimates the expression level of the respective probe set. All values are separately calculated for PM and MM probes. Their characteristics are essentially identical upon non-specific hybridization at small Σ-values. Beyond a threshold the data split into two branches due to the onset of specific hybridization. Washing removes specific transcripts more strongly from the MM owing to their weaker binding caused by their central mismatch.

In panel a of Figure 6 we plot the mean intensities (log-scale) and the washing parameters as a function of the log-mean of the PM and MM intensities averaged over each probe set (Σ, see Eq. (19)). The abscissa is governed by the concentration of specific transcripts interrogated by the respective probe set, i.e. Σ ~ [S] [10] to a good approximation. The virtually identical mean intensities of the PM- and MM-probes at small Σ-values are characteristic indicators for the predominance of non-specific hybridization of these probes because of the absence of specific transcripts. At a certain threshold of Σ (see the vertical dotted line in Figure 6) the PM and MM curves split into two branches due to the onset of specific binding. The observed intensity difference between both probe types can be simply explained by the larger binding constant of specific binding of the PM probes compared with that of the mismatched ones, i.e. K^PM,S ^> K^MM,S^.

The analogous plot of the asymptotic washing rate and of the decay constant (parts b and c of Figure 6) shows a similar split of the respective PM- and MM-characteristics. This result again illustrates the direct relation between the probe intensities and the washing parameters discussed in the previous section. The washing step removes about 90% of the initially bound non-specific transcripts (w^N^∞ < 0.1) from both the PM and MM probes. In contrast, only 10% of the specific transcripts associated with the PM-probes (w^PM,S^∞ > 0.9) but about 50% bound to the MM probes (w^MM,S^∞~0.5-0.6) are washed off. The higher limiting washing rate of the MM probes is obviously the consequence of their smaller specific binding constant, K^PM,S ^> K^MM,S ^(see above).

The washing step consequently improves the performance of the chip experiment because it removes non-specific transcripts much more strongly than specific ones [3,21]. Washing thus selectively reduces the relative contribution of the non-specific signal. Also the relative signal of the MM-probes is reduced upon washing. The vertical difference between the PM- and MM-branches at larger abscissa-values in panel a of Figure 6 indicates that washing decreases the saturation level of the MM-probes to a larger extent than that of the PM. The different saturation intensities of PM and MM probes have been observed previously and attributed to different washing rates of both probe types [9].

Note also that the limiting survival fraction w∞ (and also the characteristic washing time) monotonically increases with Σ, which suggests less effective washing at larger transcript concentrations. The observed intensity represents however the superposition of contributions due to the more stable specific transcripts and less stable non-specific transcripts (Eq. (7)). The observed trend therefore reflects the increasing contribution of specific hybridization and not the change of the individual washing rate as a function of transcript concentration. At large abscissa values the discussed washing parameters level off to their asymptotic values referring to the average values for specific transcripts (see Figure 6).

### Hybridization regimes

**Figure 7:**
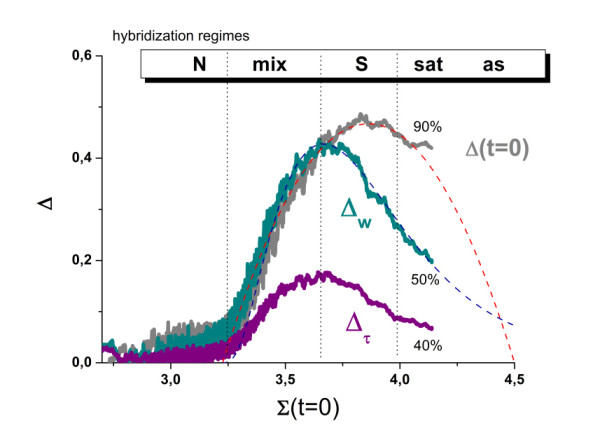
**Hook representation of the washing parameters**: The figure shows the PM/MM-difference plot of the smoothed intensities before washing (Δ(t = 0) = logI^PM^/I^MM^), of the asymptotic washing level (Δ_w _= log(w∞^PM^/w∞^MM^)) and of the decay times (Δ_τ _= -(τ ^PM ^- τ ^MM^)) as a function of the mean intensity, Σ. These hook plots reveal the typical hybridization regimes: non-specific (N), mixed (mix), specific (S), saturation (sat) and asymptotic (as) as indicated in the figure. The indicated 'percent values' estimate the degree of decrease in terms of the final level compared with the maximum. The dashed curves are theoretical fits using Eq. (20) (Δ(t = 0)) and Eqs. (37)-(38) (Δ_w_), respectively. Note that theory predicts an asymmetric shape of Δ_w _compared with the symmetric shape of Δ(t = 0) in agreement with the experimental curves.

The different hybridization regimes can be more clearly identified and characterized by direct comparison of the respective PM and MM values. The GeneChip technology uses these probe pairs of perfect matched and mismatched probes where the latter are intended to serve as an intrinsic reference for the former ones. Specific differences between the characteristics of both probe types can be extracted using a special version of the M-A-(difference-versus-sum) plot which relates the logarithmic difference of the PM and MM intensities, Δ, to their log-mean, Σ (Figure 7, see also Methods, Eq. (19) and [10,11]).

This so-called hook plot reveals details of the probe-hybridization more clearly than the log-intensity plots shown in Figure 6 (part a): The curve obtained can be divided (from the left to the right) into the non-specific (N-), mixed (mix-), specific (S-), saturation (sat-) and asymptotic (as) ranges (see also [10] for a detailed description). In the N-range the probes hybridize predominantly non-specifically. Specific hybridization comes progressively into play in the mix-range which causes the marked increase of the Δ-values and the steep increasing slope of the hook curve. The hook-curve reaches its maximum in the subsequent S-range with dominating specific hybridization of the probes. The probes progressively saturate in the subsequent decaying sat-range of the curve. Finally, it reaches the asymptotic regime referring to the maximum possible PM- and MM-intensity values.

The experimental hook curve of the unwashed chip (t = 0) is accurately described using the Langmuir binding model (see [10] for details, Methods-section and the respective dashed curve in Figure 7). We selected subsets of probes from the N-range (i.e. to the left from the break point) and from the S- and sat-ranges to calculate the intensity distributions shown in panel b and c of Figure 3 above.

The effect of washing on the position and dimensions of the hook-curve will be discussed in the next section. Here we calculate analogous PM/MM-difference characteristics for the washing parameters, Δ_w _= (log w∞^PM ^- log w∞^MM^) (Eq. (37)) and Δ_τ _= (τ^PM ^- τ^MM^), and compare them with the log-intensity difference, Δ(t = 0) = log I^PM^(0) - log I^MM^(0) (Eq. (19)) in Figure 7. The hook-curves obtained for the washing parameters partly resemble the course of the 'intensity' hook indicating the close relation between washing parameters and the intensity in agreement with the results discussed above. Note however two distinct differences: Firstly, the maximum of the 'washing' hooks is shifted to the left from the S- towards the mix-range which results in an asymmetric shape of the curve. Secondly, the end-point of the curves decay to less than 50% of the maximum value in contrast to the log-intensity hook which decays only to about 90% (see the curves in Figure 7).

The asymmetrical shape of the washing-hook can be rationalized by the fact that the PM-probes hybridize to a larger degree with specific transcripts in the mix-range than the MM-probes, the specific binding affinity of which is reduced by the mismatched pairing in the middle of their sequence. In particular, the asymptotic washing level (w∞) of the PM and MM in the mix-range is governed by the competition between specifically and non-specifically bound transcripts. The latter N-transcripts dominate the washing of the MM probes whereas the former S-transcripts dominate the washing of the PM probes. This difference results in markedly smaller values of the asymptotic washing level of the MM (and of their decay constant) and thus in relatively large Δ_w _and Δ_τ _values compared with Δ(t = 0). In the S-range also the hybridization of the MM becomes dominated by specific binding with larger asymptotic intensity levels. As a consequence the 'washing' hooks start to decrease in the S-range at smaller Σ-values than the respective intensity-hook which decays due to the onset of saturation only in the sat-range. This 'delayed' decrease of the log-intensity difference also explains its larger final level observed in the experiments.

Upon complete saturation of the probes one expects a vanishing PM/MM-log intensity difference Δ(t = 0)→0. The experimental data however indicate that the probes are not yet fully saturated. In the Methods-section we propose a simple fit equation for the w∞-hook which is based on the washing kinetics established above and on the Langmuir binding isotherm. It accurately approximates the experimental data and, moreover, allows extrapolation of the asymptotic Δ_w_-value referring to complete saturation (see the dashed curve in Figure 7). This asymptotic Δ_w_-value is inversely related to the specific binding constant of the MM-probes (Eq. (39)).

### 'Hook' characterization of washing

**Figure 8:**
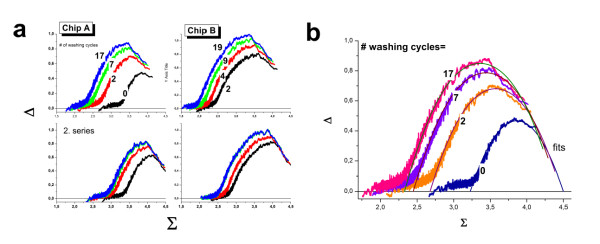
**The effect of washing on the Hook-curves**: In the second series the chips are labeled and stained a second time and subsequently washed using the same protocol as in the first series. Panel b re-plots the hook-curves for chip A (1 st series) together with theoretical functions which were calculated using Eq. (20) for different numbers of washing cycles. Washing first of all increases the width and the height of the hooks. The two trends reflect different effects: The increased width can be attributed to the strong removal of non-specific transcripts whereas the increased height indicates the stronger effect of washing on the MM-probes. Specific transcripts bound to the PM probes are relatively stable against washing as indicated by the virtually invariant right flank of the hook curves. See also Figure 17 below (part a) which assigns the geometrical dimensions of the hook-curve to the parameters used.

The hook curve provides simple overview characteristics of each hybridized chip in terms of its position (start coordinates, Σ_start_(t), Δ_start_(t); see also Eqs. (22) below) and dimensions (height and width, α(t) and β(t), respectively; see Eq. (23)). Panel a of Figure 8 shows the hook curves of the studied chips (A and B) scanned after each washing step according to the experimental protocol as illustrated in Figure 1. It turns out that washing essentially increases the vertical and horizontal dimensions of the curve. Particularly, (i) the left, increasing branch of the curve shifts markedly to the left towards smaller Σ-values whereas the right, decreasing branch and the Σ-coordinate of the endpoint remains nearly invariant; (ii) the Δ-coordinates of the maximum and of the endpoint distinctly increase whereas the Δ-coordinate of the start-point remains virtually unchanged.

**Figure 9:**
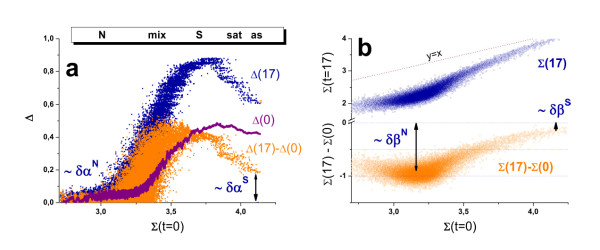
**Differential effect of washing between PM and MM probes (part a) and between specific and nonspecific binding (part b)**: Differences are calculated from the intensities after 17 washing cycles (t = 17) relative to the unwashed chip (t = 0). Panel a illustrates the Δ-coordinates Δ(0) and Δ(17) as a function of the mean intensity Σ(0) and their difference Δ(17) - Δ(0). It shows the typical hook-like shape indicating maximum washing effect on the PM/MM log-intensity difference in the mix-hybridization range. The effect is markedly reduced in the sat- and as-ranges because specific transcripts bind relatively strongly to both PM and MM, strongly reducing their washing efficiency. The PM/MM-ratio of the limiting saturation intensities is given by δα^S^~0.2 (see also Eq. (28) in the Methods-section). The difference in washing effects virtually disappears in the N-range because the weakly bound nonspecific transcripts are washed off from the PM and MM probes in nearly identical amounts (i.e. δα^N^~0). Panel b correlates the Σ-coordinates at t = 0 and t = 17. The lower plot shows the difference Σ(17) - Σ(0). The washing effect on the mean intensity is maximum in the N-range (δβ^N^~-0.95, see Eq. (27)). It gradually decreases with increasing contribution of specific hybridization to about δβ^S^~-0.13.

Figure 9 shows ordinate- (Δ, panel a) and abscissa- (Σ, panel b) coordinates of the first and last measurements at t = 0 and t = 17, and their difference to illustrate the washing effect observed in the experiment. As discussed in the previous sections, the increment of the PM/MM-log difference, δΔ = Δ(17)- Δ(0)≈ δα, virtually disappears in the N-range because PM and MM probes are equally affected by washing on the average (δα^N ^≈ 0, see also Eq. (28) below). The maximum in the mix-range simply reflects the larger amount of specific hybridization of the PM whereas the "final" level at large Σ-values is caused by the less effective washing of the PM probes due to the more strongly bound specific transcripts. This difference gives rise to different saturation levels of the PM- and MM-probes which is characterized by the mean log-intensity ratio, δα^S ^≈ 0.2.

In contrast, the increment of the log-mean intensity, δΣ = Σ(17)- Σ(0) ≈ δβ, reflects the mean washing rate of the PM- and MM-probes in the respective hybridization range (see above). The washing rate is maximum in the N-range due to the relatively weak binding of the non-specific transcripts (δβ^N ^≈ -0.95, see also Eq. (28) in the Methods-section). It progressively decreases by about one order of magnitude with increasing amount of specific binding (δβ^S ^≈ -0.13).

In summary the hybridization regimes can be associated with specific washing rates which reflect the binding characteristics of the PM and MM with the respective targets.

### Washing kinetics of hook parameters

In the previous sections we analyzed the effect of washing on the intensity of the PM and MM probes in the different hybridization ranges after the first and the last washing cycle to discover the basic changes after the washes. In this section we address in more detail the washing time dependence using all measured time points.

**Figure 10:**
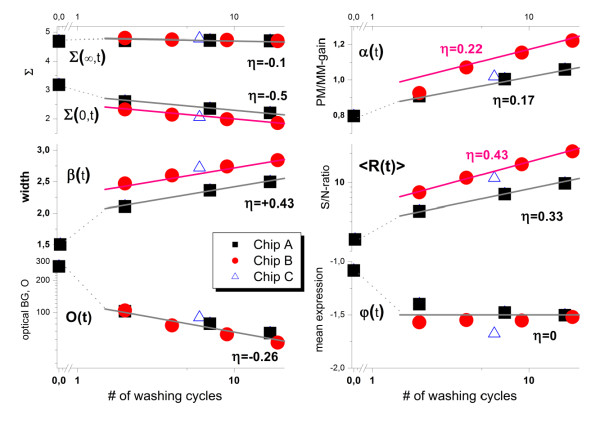
**Washing kinetics of the hook-parameters**: Hook parameters refer to the studied three chips A, B and C in the first washing series. The kinetic exponent η estimates the slope of the linear fits. The respective values are given in the figure (see Eq. (10)). Chips A and B are scanned at different time points where the first scan of chip A was performed before washing (see Figure 1). Chip C (triangle) refers to the standard washing protocol suggested by the manufacturer. The different chips provide consistent slopes within ± 0.05. The hook parameters are defined in the Methods-section.

The hook analysis provides a straightforward method to summarize the hybridization characteristics of each chip in terms of a small number of selected parameters which are obtained by fitting a theoretical hook curve to the experimental one. The fitted equation is derived by combining the two-species Langmuir adsorption isotherm with post-hybridization washing kinetics of the PM and MM probes (see Methods-section, Eq. (20)). The proposed function fits well to the experimental data obtained from the washing experiments (see panel b of Figure 8). For automatic fits in standard analysis we used the available hook program which, to a good approximation, applies also to the washing experiment (see below, Eqs. (29) - (32)). In particular, we estimated and plotted the following hook parameters as a function of the number of washing cycles t (see Figure 10):

- the mean intensity levels of saturation Σ(∞,t) and of non-specific hybridization Σ(0,t);

- the width β(t) and the height α(t) of the hook curve, defined by Eq. (23). These parameters serve as measures of the binding strength of non-specific hybridization and of the apparent PM/MM-gain, respectively;

- the mean S/N-ratio (specific-to-/non-specific hybridization strength) of the signal R(t) defined by Eq. (21). It represents a sort of mean 'signal-to-noise' ratio of the measurement;

- the mean level of specific hybridization φ(t) defined by Eq. (24). It estimates the mean expression level of all detected genes.

- In addition we also take into account the washing-time dependence of the optical background O(t) which has been subtracted from the raw probe intensities before the hook analysis as described in the methods section. The optical background is partly attributed to a residual amount of free labels which are expected to be progressively removed upon washing.

Figure 10 shows the obtained hook parameters as a function of the number of washing cycles for the studied chips A (four scans, first scan before washing), B (four scans, first scan after washing) and C (one scan only after 6 standard washing cycles). The observed kinetics can be approximated in the log-log plots for t >1 (i.e. considering all time points except the first one) by linear functions of the form(10)

(see lines in Figure 10). Note that both types of measures Ψ and F scale logarithmically with the intensity and/or binding strength: Ψ~ logF~ logI~ logX (see, e.g. Eqs. (19) and (22)). The slope-parameter η scales the washing time exponentially, i.e., (Ψ(t)-Ψ(1)) = log t^η^. The obtained slopes agree well between the independent washing experiments using chips A - C.

Note that the parameters which are related to the probe intensities (the start and end points, Σ(0,t) and Σ(∞,t), respectively; and the optical background, O(t)) decrease with washing time (η < 0) reflecting the progressive washing-off of bound targets. Contrarily, the dimensions of the hook (height and width, α(t) and β(t), respectively) and the mean S/N-ratio (<R(t)>) increase with washing (η > 0). One sees from the interpretation of the hook dimensions (see Eq. (23)) that the positive signs of the slopes reflect a stronger removal of (i) non-specific transcripts compared with specific ones (β(t)) and (ii) MM-bound specific transcripts compared with the removal of PM-bound specific transcripts (α(t)). The selective washing of nonspecific transcripts gives rise to the progressive increase of the mean S/N-ratio <R(t)>. Hence, the washes effectively improve the specificity of the expression measurement. The average signal-to-noise ratio increases roughly by the factor of 2 - 2.5 per 10 stringent washing cycles (η_R_~ 0.33 - 0.43). On the other hand, the mean expression level (φ(t), see Eq. (24)) remains virtually constant after washing (η_φ_≈ 0). We will discuss this result below.

The considered hook parameters depend on the washing functions w^P,h^(t) which characterize the removal of specific and nonspecific transcripts from the PM and MM probes (see Eqs. (21)- (26)). The direct link between the kinetics of the hook parameters (Eq. (10)) and the washing functions can be established by assuming analogous power-law kinetics of the latter ones, i.e., log(w^P,h^(t)) ~ - η^P,h^·log t (Eq. (33), see Methods-section for details). Accordingly, the three relevant exponents of the washing function can be expressed as linear combinations of the slopes of the hook parameters (Eq. (36)). With the values of the slopes given in Figure 10 one gets for the kinetic exponents of the washing functions for non-specific transcripts η^PM, N ^= η^MM, N ^= 0.5 ± 0.1, and for the washing exponents of specific transcripts of the PM and MM probes, η^PM,S ^< 0.05 and η^MM, S ^= 0.15 - 0.2, respectively. These values reflect the fact that washing basically removes weakly bound nonspecific transcripts whereas specific transcripts, especially if bound to the PM probes, remain relatively stable against washing.

The analysis using a power law (Eq. (10)) complements our simple initial analysis in terms of a two-component decay function (Eq. (8)): In particular, the power law exponent obtained enables the effect of washing to be extrapolated to times exceeding the time range of the experiment. Note that the exponent estimates the decrease of the respective washing function upon increasing the number of washing cycles by one order of magnitude, i.e. - η = log(w^P,h^(10^1^)/w^P,h^(10^0^)). With this interpretation we can estimate the number of washing cycles after which the number of bound targets is expected to reduce on the average by one order of magnitude, t* = (0.1)^-1/η^. Particularly, one gets t*~ 10^2 ^washing cycles for non-specific targets, but about t*~ 10^20 ^for specific targets bound to PM probes and still more than 10^5 ^cycles for specific targets bound to MM probes. Hence, most of the specific transcripts are virtually un-washable from the PM probes, whereas the non-specific transcripts, on average, can generally be removed by further washing.

The above power-law kinetics can be interpreted as the superposition of a large number exponential decay functions with a broad distribution of decay constants which are typically observed in heterogeneous systems with a large variety of different energetic states [22]. This description is equivalent to a time-dependent washing rate leading to the progressive slowing down of washing with increasing number of washing cycles given below in Eq. (34). The application of this interpretation to the chosen summary characteristics of the hook analysis seems reasonable because the respective parameters are mean values averaged over a large number of probes with a broad distribution of individual binding constants.

### Re-labelling (second staining/washing series)

**Figure 11:**
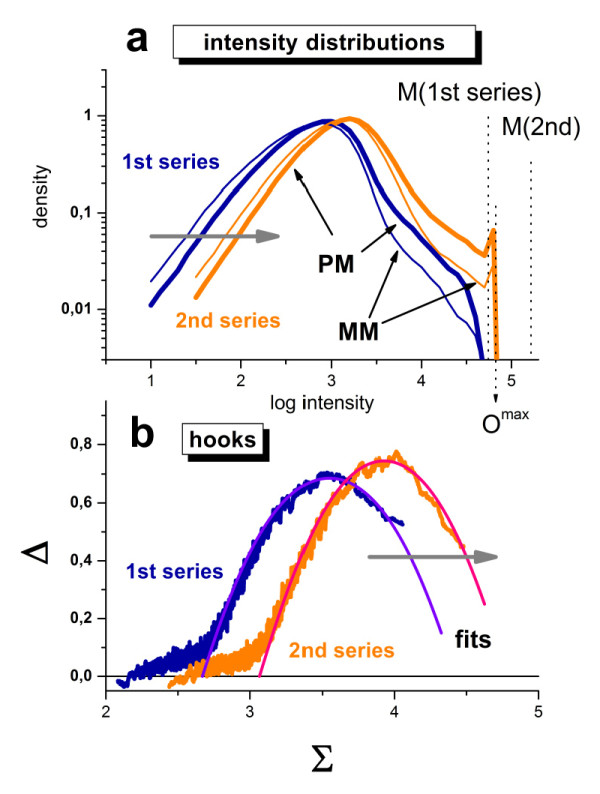
**Effect of re-labelling of array A on the total intensity distributions of the PM and MM probes (panel a) and on the hook curves (panel b)**: The data refer to the second scan A2(2x) (see Figure 1). The distributions and hook curves shift to the right after re-labelling as indicated by the grey arrows. This shift applies also to the saturation intensity: M(1^st ^series)→M(2^nd ^series). The latter value exceeds the maximum detectable intensity of the scanner, i.e. O^max ^< M(2^nd^). This constraint truncates the intensity distributions at O^max ^and sets all intensity values greater than the optical limit equal to O^max ^which causes the small peak at the right end of the distribution. Note that the width of the hooks remains virtually the same in both series whereas its height slightly increases after re-labelling. This result suggests that labelling with SAPE facilitates the washing-off of probe-bound targets. The washing-time kinetics of the hook-parameters is shown in Figure 12.

The experimental pipeline of consecutive washing cycles was repeated a second time for all three arrays A - C after completing the first washing series (see Figure 1). This second series starts with a second staining protocol before washing which consists of three alternating cycles of SAPE/anti-SAPE labelling. The additional labelling of the bound targets markedly increases the intensity level of the scanned arrays of the second series as indicated by right-shift of the intensity distributions and of the hook curves compared with the respective characteristics of the first series (see Figure 11 and also Figure 8, panel a).

**Figure 12:**
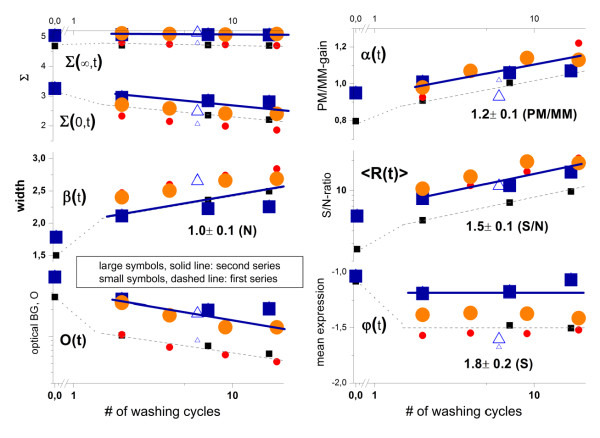
**Washing kinetics of the hook-parameters of the studied three chips before (small symbols) and after (large symbols) re-labeling**: The small symbols referring to the first series were re-plotted from Figure 10. The thin dotted lines and the thick lines serve as a guide for the eye to illustrate the trends of the first and second series (chip A), respectively. The respective enrichment factors are given in the right part of the figure (see also Figure 13). The intensity-related parameters Σ(∞,t), Σ(0,t) and O(t) shift to larger values after re-labelling whereas the width of the hook β(t) roughly agrees in both series. The vertical shift between the levels of mean expression φ(t), the mean S/N-ratio <R(t)> and also the PM/MM-gain α(t) reflect the enrichment in the second series as predicted by the simple model illustrated in Figure 13 (the numbers in the right part are the respective enrichment factors, see text).

Generally, the kinetics of the hook parameters obtained from this second series follow the same linear trends as the first series according to Eq. (10) (compare large and small symbols in Figure 12). The systematically larger values of the optical background, O(t), and of the start and end points of the hook curve, Σ(0,t) and Σ(∞,t), respectively, reflect the increased intensity level of the second series as discussed above. The very similar slopes, especially for the shift of the maximum intensity Σ(∞,t) of both series indicates that nonlinearities of the calibration curve of the scanner near its saturation level [20] can be neglected to a good approximation because otherwise one would expect to observe a decreased slope of the larger intensity values of the 2^nd ^series.

Surprisingly, the width of the hook curve, β(t), is virtually identical in both series and changes similarly with washing time. Naively it might be expected that the final washing level of non-specific hybridization reached in the first series would provide the starting level of the second series which then will be further reduced upon continuing the washes. However in contrast to this expectation that washing in the second series simply continues the first one, we observe similar widths of the hook curves in both series (see Figure 12 and also part b of Figure 11). In other words, the original level of the nonspecific background before the first washing series is actually reconstituted after re-labelling of the targets in the second staining step.

A hint for a first, tentative explanation of this result is given by the small peak at the right end of the intensity distributions of the PM and MM-intensities (see part a of Figure 11). We attribute this peak to optical saturation of the scanner giving rise to a certain fraction of probes with an essentially identical maximal intensity value, O^max^. Optical saturation leads to underestimation of the level of chemical saturation for O^max ^< M, which in turn underestimates the width of the hook and finally will overestimate non-specific binding. Optical saturation however affects only large intensity values I > O^max^. Detailed comparison of the intensity distributions of both series indicates essentially identical shapes (part a of Figure 11). The discussed shift between both series applies obviously to all intensity values over the whole intensity range and not only to large intensities in the saturation range of the scanner.

**Figure 13:**
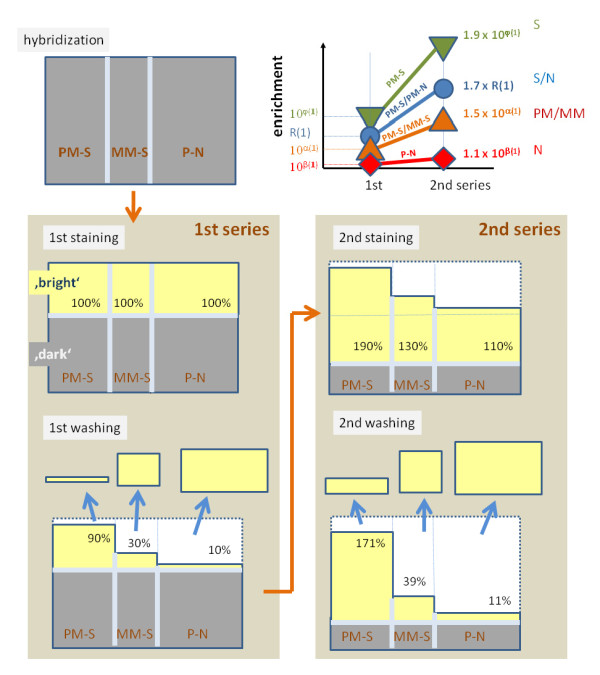
**Schematic illustration of the effect of washing of the microarray using two rounds of staining and washing**: The different steps are characterized as follows: **Hybridization**: The grey areas refer to the amount of PM and MM probe oligomers occupied with specific (S) and nonspecific (N) transcripts (see Eq. (1)). Both probe types are assumed to hybridize identically with non-specific transcripts (P-N, P = PM,MM). Free probe oligomers are not shown. **1**^**st **^**staining**: A certain fraction of bound transcripts becomes 'bright' by labeling with fluorescent markers (SAPE) whereas the remaining non-labeled fraction remains 'dark'. The amount of bright probe duplexes of each type in this first labeling round is set to 100%. **1**^**st **^**washing**: Washing removes bound targets of the bright fraction from the probes as indicated by the arrows (Eq. (4)). The yield of washing depends on the duplex type: The percentage of reduction of bound targets is largest for nonspecific transcripts and smallest for specific transcripts bound to PM probes. The dark fraction is not affected by washing. **2**^**nd **^**staining**: We assume that in the second staining round the same amount of dark probes is transferred into bright ones by labeling as in the first round. The given percentages refer to the amount of bright probes relative to the initial level after 1^st ^staining. For example, the amount of bright PM-S duplexes nearly doubles from 90% to 100%+90% = 190%. **2**^**nd **^**washing**: The amount of bound targets reduces by the same duplex-specific factor as in the 1^st ^washing round. For example, 90% of the 190% bright PM-S remain bound (0.9·190% = 171% of the initial level). Note that each staining/washing round enriches high-affinity bright duplexes compared with low-affinity bright ones, e.g. PM-S compared with MM-S and with P-N as indicated in the graph in the upper part of the figure. The given enrichment factors for the expression degree PM-S and the ratios PM-S/MM-S and PM-S/PM-N refer to the second round compared with the first one (see text). Note that the respective hook parameters are related logarithmically to the enrichment factors.

We therefore suggest a second, alternative explanation which is schematically illustrated in Figure 13. In particular, we assume that the fluorescent markers bound to the targets drastically increase the washing yield of labelled transcripts compared with non-labelled ones. Note that only a certain minor fraction of bound targets becomes fluorescently labelled with SAPE, whereas the remaining major fraction remains unlabelled and does not actually contribute to the measured probe intensity [23]. SAPE represents a bulky, water-soluble protein-dye complex which covalently binds to the biotins attached to a small fraction of cytosines in the target-sequences. The molecular weight of SAPE of about ~300 kDa [24] by far exceeds that of unlabelled RNA-target fragments (e.g., ~30 kDa for target lengths of 100 nucleotides). Hence, properties of the SAPE/RNA complexes such as molecular weight, size and hydrophilicity are expected to be dominated by the SAPE component. It is well established that the nature and position of attached fluorescent labels affect the signal intensity of surface-bound probe/target duplexes [25]. Our proposed explanation is that the SAPE-markers drastically reduce the resistance to dissociation by washing of labelled targets compared to that of non-labelled ones. As a consequence, washing is expected to remove essentially only the labelled targets whereas non-labelled dark ones remain bound to the probes. In the second series, a certain fraction of these dark targets becomes labelled and thus visible after repeated staining of the chips.

For a simple estimation of the enrichment after two rounds of staining/washing we set the initial amount of labelled bright targets in each type of duplexes to 100% and assume duplex-specific washing yields in rough agreement with our analysis (see also legend of Figure 13). We also assume that the same amount of dark transcripts is transferred into bright ones in both staining rounds. This approximation applies for a large excess of dark transcripts. About 90% of the specific transcripts bound to the PM probes consequently survive the first washing round. Then, relabeling increases the total amount of bright targets again to 190% compared with the result of the first labelling round. The assumed washing yield of 90% reduces the final amount of bright targets to 190%·0.9 = 171% after the second washing round. The bright specific targets bound to the PM probes enrich consequently by a factor of about 171%/90% = 1.9 compared with one staining/washing round. Analogously one can estimate the enrichment factors for the different duplexes and their ratios. They decrease according to PM-S > PM-S/P-N > PM-S/MM-S > P-N (see the schematic plot in Figure 13). The nonspecific background only weakly enriches by a factor of 1.1, which is hard to observe experimentally. Indeed, we found similar nonspecific background levels β(t) in both series. Comparison of the hook parameters φ(t), <R(t)> and α(t) of both series provides experimental estimates of the enrichment factors (see Figure 12, right part; note the logarithmic scale). Importantly, the values obtained rank in the same order as that predicted by our simple model.

### Sequence effects

In early papers the washing efficiency of surface-bound probe/target duplexes was used to estimate the stability of selected sequence motifs in terms of thermodynamic parameters such as the free energy of probe/target association [12]. Two high- (labels a and b) and two low-intensity (c and d) probes are labelled in Figure 2 together with their sequences and the respective total numbers of nucleotides A, C, G and T per sequence. One of the high-intensity probes (label a) contains eight cytosines, five of them are assembled in runs of two and three adjacent Cs. These motifs are associated with high binding affinities and thus weak washing potential (see also below). The overall base compositions of the remaining probe sequences (b - d) however look essentially similar although their intensities differ by up to three orders of magnitude.

**Figure 14:**
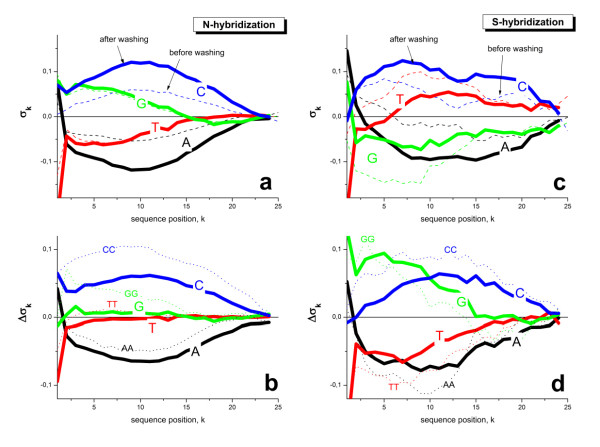
**Positional sensitivity profiles of specific (panel a) and non-specific (panel c) hybridization before (t = 0, thick curves) and after (t = 17, thin dashed curves) washing**: The respective nucleotide-letters are given in the figure. The respective sensitivity terms estimate the mean contribution of the selected nucleotide at the given position to the observed intensity-increment with respect to the set-mean of the intensity (log-scale, see Eq. (41)). The two panels below (b and d) show the difference profiles 'washed-unwashed'. In addition to the single-base terms we also show nearest-neighbor (NN) terms of selected homo-couples (dotted curves, the original NN-profiles in the upper panels are omitted for clarity). The difference profiles estimate the mean relative stability of the respective nucleotide-letter at the given position against washing. Note that sequence position k = 1 faces towards the bulk solution whereas position k = 25 is attached to the chip-surface.

To extract the relation between the probe sequence and washing efficiency in more detail we make use of the positional dependent sensitivity model which, in its simplest version, estimates the mean contribution of each nucleotide letter at each sequence position to the probe intensity [10,26,27] (see below, Eq. (41)). These so-called sensitivity profiles were separately calculated either for probes which are predominantly hybridized with non-specific or with specific transcripts before and after washing (see panel a and c of Figure 14). In addition we calculated the respective difference-profiles 'after washing-minus-before washing' to extract the effect of washing on the profiles (panel b and d of Figure 14).

The non-specific profiles of nucleotides A and C show the typical parabola-like shapes with their minimum/maximum values near the center position of the probe sequence (see, e.g. [26,28]). Washing inflates these curves (compare dashed and solid curves in part a of Figure 14) indicating that the specific sequence effect of the different nucleotides on the intensity increases after washing. In particular, the difference profiles show that the intensity-difference between cytosine-rich and adenine-rich probes increases with washing. This trend can be easily rationalized by the stronger base pairings formed by the C's. They cause not only larger intensities of C-rich probes before washing [15], but also their stronger resistance against washing. As a consequence the base-specific effect increases with washing. The observed trend is supported by theory (see Eq. (45) in the Methods-section).

The shape and the values of the specific profiles differ from the profiles of the non-specific ones (compare dashed curves in panel c and a of Figure 14). This change can be explained by the stronger effect of saturation on the intensities [29] and/or by bulk hybridization of the specific transcripts [30]. Interestingly, the difference profiles closely resemble that of non-specific hybridization (compare panel d, b and a of Figure 14). These results imply that the base-specific efficiency of washing is less distorted by saturation and bulk hybridization than the binding affinity of the transcripts. This interpretation seems reasonable because saturation and possible bulk effects will not affect the removal of bound transcripts according to the respective reactions (Eq. (4)). In the Methods-section we link the sensitivity profiles, σ_k_, and their increment upon washing, Δσ_k_, with the base and positional dependent affinity of base pairings, ε_k _(Eqs. (45) and (46)). According to Eq. (46), washing is indeed expected to expose the sequence effects of direct probe/target interactions, i.e. Δσ_k_∝ ε_k_, which are partly distorted in the original sensitivity profiles due to specific hybridization (see Eq. (45)).

In addition to the single-base sensitivity profiles we apply the positional-dependent nearest neighbour sensitivity model to the intensity data. Part b and d of Figure 14 show the incremental-profiles of the homo-couples AA, TT, GG and CC (dashed curves). Particularly, the CC-profiles significantly exceed that of single C: Hence, two neighbouring cytosines more strongly resist washing than, e.g., two cytosines which are separated by other intervening bases.

In summary, we found that washing (i) increases the sequence-specific sensitivity of the probes for target binding and (ii) decreases the effect of saturation and bulk hybridization on the observed profiles. The resulting sensitivity profiles depend directly on the strength of the probe/target interactions and on the washing function which, in turn, is an exponential function of the probe/target interactions.

## Discussion

### The washing rate is intensity- and time-dependent

We experimentally explored the effect of repeated stringent washing cycles on the hybridization signals of GeneChip microarrays and analyzed the observed intensity changes in the framework of accepted hybridization models. The washing rate is intensity and time-dependent. We found that

(i) the efficiency of washing after a given number of washing cycles is decreased with the probe intensity, i.e. the relative intensity decrement of low intensity probes after washing is much larger than that of high intensity probes.

(ii) the effective washing rate is not a constant. Instead, it decays with progressive washing meaning that the intensity decay upon washing is not a single-exponential one. In particular, the first few stringent washing cycles after hybridization give rise to a much larger intensity decrement than the following washing cycles which only moderately affect the probe intensities.

The first result (i) can be readily explained if one assumes that the washing efficiency is exponentially related to the affinity constant of probe/target association which, in turn, governs the signal intensity. The resulting power law of the binding constant gives rise to an intensity threshold which separates low-intensity probes prone to washing from higher-intensity probes which strongly resist washing.

The measured probe intensity is the superposition of two distinct contributions due to non-specific and specific hybridization. The respective targets are characterized by relatively small and high values of the binding constant, respectively. Specific binding to the MM probes earns an intermediate binding constant due to a single mismatch at the center position of the sequence. As a consequence, stringent washing of GeneChip arrays in the window of 17 washing cycles used selectively removes, on the average, more than 90% of the non-specific fragments from the probes. In contrast, only 50%-30% of the specific targets are removed from the MM-probes and less than 10% from the PM-probes. The different washing efficiencies of the PM and MM probes for specific transcripts gives rise to larger asymptotic intensity levels of the PM-probes compared with the MM-probes (see Eq. (25)).

The average effect of washing is modulated by the individual probe sequences giving rise to a wide spread of probe-level values. For example, runs of adjacent cytosines stabilize probe/target duplexes against washing on a relative scale whereas adenines facilitate dissociation of the duplexes. The sequence-dependence of the washing rate causes an increasing sequence-dependent variability of the probe intensities with progressive washing.

The second result (ii) can be attributed to the heterogeneous character of probe/target-binding owing to, e.g., different sequences enabling non-specific association to a given probe and/or to non-equilibrium zippering effects which give rise to a series of different microstates states for the same probe/target duplex (see e.g. [4] and below). Simply speaking, weakly-bound duplexes dissociate faster and become depleted from the heterogeneous ensemble of binding-states. On the other hand, the proportion of strongly-bound duplexes becomes enriched with washing, giving rise to a decrease in the mean washing rate. Our empirical analysis is consistent with a washing rate which decays hyperbolically with the number of applied washing cycles (Eq. (34)).

Re-labelling of previously washed arrays and a subsequent second washing round reveals that the bulky hydrophilic SAPE markers strongly facilitate washing. In consequence, additional re-labelling/washing rounds strongly enrich the ratio of bound specific duplexes to weakly bound complexes. Re-labelling however runs the risk of optical saturation of a fraction of the probe spots because it markedly increases the intensity level of the array.

The primary purpose of our study is to quantify and correct for the systematic effects of post-hybridization washing. Stochastic variability is accounted for by the O(10^5^) features on each microarray. In principle therefore, technical replicates of the entire array under each washing protocol are unnecessary provided that each hybridization satisfies minimum quality and reproducibility criteria which have been proven using the hook analyses (see Figure 8 and next subsection). Furthermore, we note that the three arrays considered are identical with respect to chip type, hybridized RNA extract and hybridization protocol, the only difference being mutual changes in washing times between scans. Figure 10 clearly shows a consistent behaviour across chips: the slopes obtained for experiments A and B agree to within 10-25%, roughly corresponding to the regression error. Similar outcomes were obtained in a pre-experiment with two technical replicates of GeneChip Test3 arrays (see additional file 1: Supporting text).

### Hook-curve analysis as a tool to study washing

The so-called hook method uses the MM-probes as an internal reference to calibrate the intensities of the PM-probes with respect to the non-specific background and with respect to the saturation level of the probe spots at small and high intensities, respectively. In its original 'standard' version, the method assumes target binding according to the hyperbolic Langmuir adsorption isotherm which neglects the washing step [10,11]. The method can be easily extended by assuming appropriate washing functions (see Methods-section), to give a modified version which fits the experimental washing data.

In the first instance, the hook method aggregates the hundred thousands of intensity data per chip into a few key parameters which characterize the hybridization of the particular array and their changes caused by washing. Besides data-reduction, a second advantage of the hook method lies in the geometric meaning and interpretability of the model parameters: The position and dimensions of the hook-graph are directly related to essential hybridization characteristics such as the level of non-specific hybridization, the PM/MM-gain and the saturation intensity.

We show also that the 'standard' hook method based on the Langmuir adsorption law readily applies to post-hybridization washing data. The physical meaning of the obtained hook parameters is partly modified: For example, the estimated levels of nonspecific background and of saturation, and the PM/MM-gain refer to hybridization *and *subsequent washing whereas its original meaning included only the hybridization step.

One consequence of this changed interpretation is to the results of our recent study addressing the relation between the nonspecific hybridization level and the specific binding constant of the microarray probes assuming equilibrium thermodynamics [31]. The respective hook estimates were used to correct the expression estimates for the so-called up-down effect, according to which an increased background level decreases the sensitivity of the probes for specific transcripts and vice versa. However, the hook method estimates the level of non specific background after washing which is markedly smaller than that immediately after hybridization. In consequence, the extent of the up-down effect is actually underestimated by ignoring the washing step. A modified correction for the up-down effect will be given elsewhere.

To illustrate the benefits of the hook approach in an independent application we transform the intensity-data of a previous washing experiment [4] into hook-graphs (see supplementary material). Despite the different design of the experiment which uses selected spikes, the respective hook data undergo virtually the same changes as in our study. Importantly, the results agree quantitatively with ours: the observed shift of the N-range, δβ^N^~0.8, agrees in both experiments despite the different chip types used by us (Human HG-U133plus2 array) and Skvortsov et al. (Drosophila DG-1 array).

These examples demonstrate that the hook-presentation of microarray data allows the simple and straightforward characterization of the effect of washes on the degree of probe/target binding. Note that our approach does not require selective labeling of spikes to differentiate non-specific from specific hybridization.

In particular, the hook curve analysis allows the effect of washing on the different hybridization modes to be disentangled for the heterogeneous ensembles making up the of hundreds of thousands of microarray probes. The time dependence of the extracted intensity-related hook parameters is given approximately by a linear function of the logged washing time. The resulting kinetic exponents η can be used to characterize the mean washing efficiency of the different hybridization modes. Washing affects non-specific binding most efficiently (η≈0.5), is intermediate for specific hybridization of the MM-probes (η≈0.15) and minimum for specific hybridization of the PM-probes (η < 0.05). This agrees with the results presented in the previous paragraph. The different values of the kinetic exponents for non-specific and specific hybridization predict that the signal-to-noise ratio (S/N-) ratio of the measurement improves by a factor of two for every ten washing cycles.

### Washing scaling of expression estimates

Rearrangement of Eq. (7) provides the specific binding strength as a function of the probe occupancy which, in turn, is directly related to the measured probe-intensity:(11)

Eq. (11) represents the basic calibration equation which inverts the adsorption isotherm and corrects the measured intensity for the parasitic effects of nonspecific hybridization, saturation and post-hybridization washing.

An estimate of the binding strength which neglects washing is given by Eq. (11) and the condition w^P,h^(t) = 1,(12)

This situation refers to the competitive two-species Langmuir-type binding behaviour which has been previously used for calibrating microarray data [10].

Note that essentially all parameters (except M) used in Eqs. (11) and (12) depend on the binding constants K^P,h ^in a sequence- and thus probe-specific fashion. For estimating the effect of washing on expression estimates we will assume t = const and K^P,h ^= const which applies, e.g., to one probe-sequence or to mean values of the binding constants averaged over all relevant probes. From these assumptions follows w^P,h^(t) = const (Eqs. (5) and (6)) and X^P,S^~[S] (Eq. (3)). Hence, the change of the specific binding strength directly reflects the change of the expression degree in this case.

**Figure 15:**
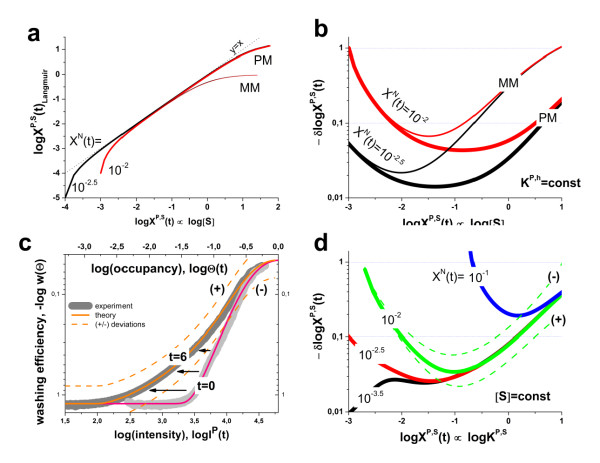
**Systematic bias of 'Langmuir'-expression estimates (Eq. (12)) with respect to the estimates which consider washing (Eq. (11))**: Part a and b: Correlation plot between both estimates and their logged difference, . The graphs were calculated assuming K^P,h ^= const for two non-specific background levels (red and black curves) and for PM and MM probes (dotted and solid curves) assuming the survival fractions w^PM,S^(t) = 0.95, w^MM,S^(t) = 0.50 and w^P,N^(t) = 0.1. Neglecting washing underestimates the expression degree especially at small and large expression values. Part c: The survival fraction of bound probes depends on the intensity (or, equivalently, probe occupancy) before (t = 0) and after washing (t > 0). The graph for t = 0 was re-plotted from Figure 5a using Eq. (17) with w_max _= 0.9, w_min _= 0.06, γ = 1.6 and a' = 0.1. The graph for t = 6 refers to the standard number of washing cycles. It is obtained from the t = 0 graph by making the substitution logI(t) = logI(0)+log(w(t)) in the argument (see Eq. (8)). Part d shows the bias of the Langmuir approximation assuming a constant transcript concentration and variable K^P,S ^and thus a variable survival fraction w(Θ) which has been taken from part c of the figure for t = 6. The dashed curves labelled with '(+)' and '(-)' in panels c and d refer to 50%-deviations of the washing function, log w(Θ)^+/- ^= log w(Θ)·(1.5)^(+/-)1^, to estimate the effect of the scattering of the probe level data from the mean (compare with Figure 5a). The bias of the Langmuir-approximation strongly resembles that shown in part b. Note that the bias applies to PM and MM probes as well in this case.

Figure 15 (part a) shows the correlation plot between the Langmuir-approximation (Eq. (12)) and the 'true' expression degree taking into account washing (Eq. (11)). The respective binding strengths were calculated for PM and MM signals at two non-specific background levels assuming reasonable washing values given in the legend of the figure. Note that the MM probes can be interpreted as a second kind of PM probe, but with weaker specific binding affinity.

The chosen range of binding strengths -4 < logX^P,S^(t)_Langmuir _< 1 refers to typical hybridizations of GenChip arrays with specific transcripts in the concentration range 10^-2^pM < [S] < 10^+3^pM [11]. The obtained bias, δ log X^P,S ^(t) = log X^P,S^(t)_Langmuir _- log X^P,S^(t), is always negative (see part b of Figure 15). Hence neglecting washing underestimates the true binding strength. The bias markedly inflates in the limit of small and large abscissa values because of the dominating effect of nonspecific background and saturation, respectively.

The bias can be rationalized if one recalls that the washing functions w^P,h^(t) < 1 effectively decrease the effect of saturation compared with the Langmuir-isotherm. Neglecting washing therefore overestimates saturation of specific and nonspecific hybridization. The bias increases at low expression levels due to the strong effect of the nonspecific background correction. At high expression degrees the bias increases because of the saturation due to specific transcripts is modified by washing.

The bias depends on the value of w^P,S^(t) which is different for the PM and MM probes due to their different binding constant for specific transcripts: The stronger washing yield of the MM, for example, gives rise to the steeper increase of the bias than the respective bias of the PM. This result is plausible, since the apparent saturation level of the MM at large binding strengths requires stronger correction than that of the PM. At intermediate values of the specific binding strength the bias is relatively small because saturation and the N-background only weakly affect the measured intensity. Note that the intermediate values refer to the S-range identified above in the hook-plots.

In summary, the Langmuir-approximation underestimates the expression degree in concentration units. More importantly, expression estimates are scaled non-uniformly relative to the Langmuir estimates without washing, the bias being most pronounced at small and large expression values. The effect may be small in an intermediate expression range over roughly one order of magnitude which is relevant for most of the transcripts.

### Correcting expression estimates for washing effects

Basically, probe intensities affected by post-hybridization washes can be properly transformed into specific binding strengths by using the calibration equation Eq. (11). Its application requires knowledge of the intensity level of saturation, M, of the level of non-specific hybridization after washing, X^P,N^(t), and of the two washing functions characterizing the removal of specific and non-specific transcripts, w^P,S^(t) and w^P,N^(t), respectively.

Our analysis shows that the probes of largest intensity of the chip almost completely resist washing (see, e.g. Figure 3). If one assumes, as a rule of thumb, that the most intense PM probes are saturated by transcripts, then their mean logged intensity estimates log M_max _≈ < log I^PM ^>_max _to a good approximation. We found that a number of ~20 - 100 probes are sufficient. The assumption that these probes are saturated can be simply proved by comparison of M_max _with the mean asymptotic intensity M estimated by the hook analysis (Eq. (23)). Typically one gets M_max _> M where M is of the same order of magnitude as M_max_.

The non-specific background contribution of each probe can be estimated using the hook method [10]. In particular, it combines the mean non-specific background level with the probe specific increment, log X^N^(t)=⟨log X^N^(t)⟩_N-probes _+ δlogK^P,N ^in analogy with Eq. (40). The mean background level is given by the horizontal width of the hook curves between its start point and the logged saturation intensity, ⟨log X^N^(t)⟩≈-β_max _- Σ^start^(t) (see also Eq. (23)). The incremental term is estimated using the positional-dependent sensitivity model,  (Eq. (41)).

For estimating the washing functions we make use of Eq. (17) which provides the survival fraction as a function of the initial probe intensity I(0) before washing (see also Figure 5a). However, the typical microarray experiment measures the probe intensities of the washed chips after t = 6 washing cycles if one applies the standard protocol. We therefore transform the argument in Eq. (17) according to log I(t) = log I(0)+log w(t). Figure 15c compares the survival fractions as a function of I(0) and I(t = 6). The shift between both functions simply reflects the intensity-dependent washing rate discussed above. After transforming the intensities into probe occupancies (Eq. (11)) one gets the general form of the washing function which applies to all probe-level data independent of the probe type (PM or MM) and of the hybridization mode (specific or non-specific) for a selected number of washing cycles, i.e. w^P,S^(t), w^P,N^(t)→w(Θ).

Taking together, the calibration equation Eq. (11) can be re-written in the following form(13)

where the occupancy of the probes due to non-specific hybridization, Θ^N^, neglects saturation. Eq. (13) applies, for example, to probes of one probe set which interrogate one transcript. We calculate the systematic bias of the respective Langmuir-estimates (Eq. (12)) for this situation assuming constant specific transcript concentration, [S] = const. The Langmuir correction underestimates the 'true' binding strength in a very similar fashion as in the special case of K^P,S ^= const discussed in the previous subsection (compare part b and d of Figure 15). Hence, the particular form of the washing function (using, e.g., the function w(Θ) or, alternatively, the fixed values w^P,S ^and w^N^) relatively weakly affects the obtained bias and thus also the specific binding strength after calibration. This result suggests that Eq. (17) together with the parameter estimates of this study (see legend of Figure 15) can be used for the washing correction of GeneChip microarray data in general. This conclusion is supported by our hook analysis of the independent washing experiment of Skvortzov et al. [4] which provides similar washing yields to our study despite the different chip types and particular realizations of the experiments.

Finally, the obtained probe-level data of the specific binding strengths can be corrected for sequence specific binding constants using the positional dependent sensitivity model in analogy with the correction of the non-specific background (see Eq. (40) and also [10]).

Note that the condition of constant transcript concentration is not strictly fulfilled by choosing the probe occupancy as argument of the washing function in Eq. (13) because the variability of the intensity is partly caused by changing transcript concentration. We estimated this effect by assuming a funnel of positive and negative deviations of the washing function which roughly covers the scattering range of the probe-level data (see the dashed curves in Figure 15c; compare with the grey dots in Figure 5a). Clearly this uncertainty can be judged as a second order correction of the basal washing effect (see Figure 15d). It can be taken into account in further amendments of the approach which, for example, correct the occupancy Θ by the expression degree of each probe.

## Conclusions

Washing deforms the equilibrium adsorption isotherm of microarray probes in a sequence dependent manner. In consequence it scales expression measures which are derived from the probe intensities. The washing effect can be taken into account by two washing functions per probe which characterise the survival of non-specific and specific probe/target duplexes after washing. We propose an empirical washing function depending on the respective intensity contribution due to specific and non-specific hybridization which can be estimated using a previously developed calibration method for microarray intensities. On a relative scale, consideration of the washing effect will increase expression measures especially in the limit of small and large values. In this publication we presented the basic theoretical framework and its experimental verification. Applications to expression analyses will be presented elsewhere. Importantly, we provide an experimental 'washing data set' which might be used by the community for developing appropriate corrections methods.

## Methods

### RNA preparation and microarray measurements

Total RNA was isolated from the human follicular cancer cell line (FTC 133) using TRIzol reagent (Life Technologies, Gaithersburg, MD, USA) according to the manufacturer's instructions. Afterwards, the total RNA was purified with RNeasy columns (Qiagen, Hilden, Germany) according to the RNA clean-up protocol. Before microarray analysis RNA integrity and concentration was examined on an Agilent 2100 Bioanalyzer (Agilent Technologies, Palo Alto, CA, USA) using the RNA 6.000 LabChip Kit (Agilent Technologies) according to the manufacturers instructions.

Microarray measurements were conducted at the microarray core facility of the Interdisziplinäres Zentrum für Klinische Forschung (IZKF) Leipzig (Faculty of Medicine, University of Leipzig). 5 μg of total RNA were used to prepare double-stranded cDNA (Superscript II, Life Technologies, Gaithersburg, MD USA) primed with oligo-dT containing a T7 RNA polymerase promoter site (Genset SA, Paris, France). cDNA was purified by phenol-chloroform extraction before in vitro transcription using the IVT labeling kit (Affymetrix, Santa Clara, CA, USA) to synthesize cRNA. After the in vitro transcription, unincorporated nucleotides were removed using the RNeasy kit (QIAGEN, Hilden, Germany) and the cRNA was fragmented. Washing and staining were done with an Affymetrix Fluidics Station FS400. All arrays were scanned with a third generation Affymetrix GeneChipScanner 3000 with the 7 G upgrade.

### Probe-level washing kinetics

The probe-level washing kinetics have been analyzed using a two-parameter empirical decay function, Eq. (8). The inferred decay time τ and the limiting washing level w∞ characterize the decays at short and long washing times, respectively. The estimated values are observed to correlate well with the initial intensity level I(0) (see Figure 5). The purpose of this section is to provide a theoretical explanation of the functional dependence of these two parameters on the initial intensity before washing, based on the kinetics of hybridization and washing at the level of individual probes. The idea behind the explanation is that the initial intensity is a function of the association constant of the respective probe/targets duplexes (Eq. (3)), which, in turn, determines their dissociation rate (Eq. (7)).

The empirical decay function Eq. (8) is a proxy for the general case of a superposition of a large number exponential contributions arising from a number of hybridized species and binding configurations with varying decay rates. To simplify matters we will concentrate here on those probes dominated by a single hybridised species whose washing function can therefore be approximated by a single decay mode exp(-k·t), in accordance with Eq. (5). We define the following empirical function to describe the washing yield for a given number of washing cycles as a function of the dissociation rate:(14)

where two new parameters, the limiting washing levels w_min _and w_max_, estimating the minimum and maximum survival fractions observed in the experiment have been introduced. Eq. (14) thus accounts for the fact that complete wash-off (w(k) = 0) and no-wash (w(k) = 1) is not observed in the data, presumably because of effects such as the limited resolution of the intensity measurement (e.g. due to the imperfect optical background correction) and the heterogeneous character of target binding.

To estimate the washing rate k in Eq. (14) note that our assumption of a single hybridisation mode implies that either only specific or non-specific binding dominates probe hybridization. The two-species hyperbolic intensity function, Eq. (7) thus simplifies to a single binding constant and target concentration with h = S or N:(15)

Rearrangement with respect to K^P,h ^and insertion into Eq. (6) provides the rate-constant of washing as a function of the probe intensity(16)

Insertion into Eq. (14) provides(17)

where a' = a·t^1/γ^, w_min _and w_max _are adjustable parameters.

The limiting washing level w∞ and the short decay time τ introduced in Eq. (8) were estimated using Eq. (9) for the number of washing cycles t = 17 and t = 2, respectively. Eq. (17) then provides the following theoretical expressions for estimating both parameters as a function of probe intensity I = I(0),(18)

**Figure 16:**
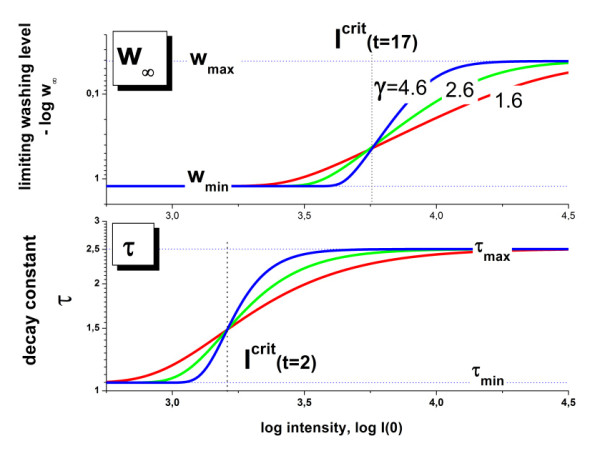
**Sigmoidal "switch"-function governed by the exponential power law of the binding constant (Eq. (16))**: The "switch"-functions are used to describe the intensity-dependence of the asymptotic washing level and the characteristic decay time (Eq. (18), see also Figure 5). The parameters "switch" between their minimum and maximum values at the characteristic intensity I^crit^≈a'M. It depends on the number of washing cycles used for parameter estimation (see Eqs. (17) and (18)). The limiting decay times are given by τ_min _= 2/ln(w_min_(2)) and τ_max _= 2/ln(w_max_(2)) (see Eqs. (18) and (17)). The sharpness of the step is governed by the exponent γ (see figure).

Eq. (18) predicts a sigmoidal change of the limiting washing level and of the decay time with increasing probe intensity (see Figure 16). The sharpness of the sigmoidal step is governed by the exponent γ (see the different curves in Figure 16). Its position is independent of the exponent γ and refers to a survival fraction of 1/e≈0.37. It is given to a good approximation by the critical intensity I^crit^≈ a'·M~ t^1/γ ^, if one neglects saturation (I(0) < < M). Hence, the scaling parameter a' essentially determines the position of the sigmoidal step along the intensity axis. It depends on the washing time (Eq. (17)). Hence, the critical intensity is different for the step functions of w∞ and τ because the respective parameters are determined for different numbers of washing cycles (see Figure 16).

### Hook analysis explicitly considering washes

The so-called hook method aims at characterizing the hybridization of a particular microarray in terms of quality control and expression analysis (see [10,11,32] for a detailed description). This single-chip method applies to microarrays of the GeneChip-type containing pairs of perfect match (PM) and mismatch (MM) probes. The method processes the PM and MM probe intensities (I^PM ^and I^MM^, respectively) using the transformations(19)

where <...>_set _denotes averaging over each probe set of usually 11 PM/MM probe pairs addressing the same transcript (log ≡ log_10 _is the decadic logarithm). Plotting the data into Δ-versus-Σ coordinates and subsequent smoothing provides the hook curve which enables decomposition of the probe signals into contributions due to specific and non-specific hybridization by simple graphical analysis and subsequent correction of the intensities for sequence specific effects using the positional-dependent nearest neighbour model as standard. The corrected signals are re-plotted into Δ-versus-Σ coordinates and again smoothed to obtain the corrected version of the hook curve.

The two-species Langmuir hybridization isotherm predicts a theoretical hook-curve which was previously used to fit the experimental curves and to extract characteristic chip-related parameters. Here we will modify the hook formalism and explicitly take into account the effect of washing: Insertion of Eq. (7) with P = PM and MM into Eq. (19) provides the theoretical expressions of the (washing) time-dependence of the hook coordinates after rearrangement(20)

with the saturation terms.

**Figure 17:**
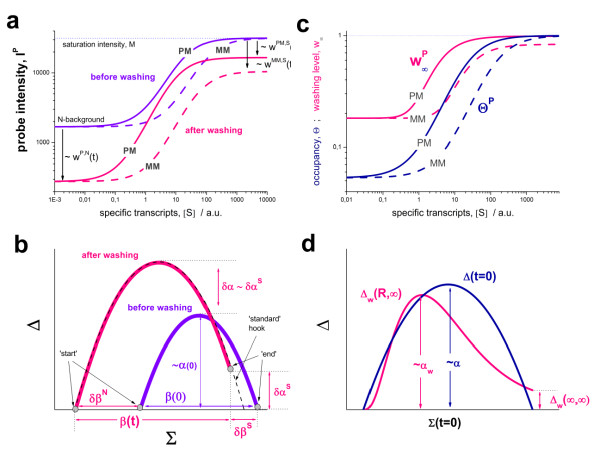
**Concentration dependence and hook representation of the washing effect**: Part a and b: PM- and MM-probe intensities before (t = 0) and after (t > 0) washing (part a) and the respective hook plots (part b, Eq. (20)). The initial intensities in the limit of small and large specific transcript concentrations decrease by the 'survival' factors w^P,h^(t) (P = PM,MM; h = N,S) after washing. These trends transform into a 'deformation' and shift of the hook curve: Washing increases its the height (α) and the width (β) by the increments δα≈δα^S ^and δβ = δβ^S^+δβ^N^, respectively (see Eqs. (23), (27) and (28)). The 'start' (R = 0) and 'end' (R = ∞) coordinates are indicated in the figure. The dashed curve is the 'standard' hook approximation (Eq. (30)). Part c and d: Occupancies of the PM- and MM-probes before washing and the respective limiting survival fractions (part c) and the respective log-intensity hook (Δ(t = 0), Eqs. (19) and (20)) and hook-presentation of the asymptotic washing level (Δ_w_(R,∞), Eqs. (37) and (38); part d). Note the asymmetric shape of the latter curve and its limiting height at R→∞ (Eq. (39)). The height-parameters α_w _and α are related to different hybridization characteristics, namely the non-specific binding strength and the PM/MM-gain, respectively (see text).

For the detailed derivation of the hook equation in the absence of washing and the detailed discussion of the used parameters see refs. [10] and [11]. The 'washing' hook is obtained analogously. Typical examples of the hook curves for t = 0 and t > 0 are shown in Figure 17.

Eq. (20) expresses the hook-coordinates as a function of the washing time t and of the washing dependent S/N-ratio,(21)

It serves as a measure of the expression degree, [S], given in units of the non-specific binding strength, the specific binding constant and of the ratio of the respective washing functions.

The course of the theoretical Δ-versus-Σ plot (Eq. (20)) is governed by four parameters with well-defined geometrical meanings (see Figure 17 and Eqs. (22) and (23) below): The "start" coordinates,(22)

and the vertical and horizontal dimensions(23)

are functions of the binding constants, the washing functions and of the saturation intensity, M(t). These parameters decompose into an initial value referring to the hook curve before washing at t = 0, and into an incremental term which explicitly considers the effect of washing. The time dependence of the maximum intensity, b(t), is attributed to bleaching. It identically affects the labelling of specific and non-specific transcripts. The approximations on the right hand-sides of the equations assume identical nonspecific hybridization and bleaching of the PM and MM probes. The 'dimensions' define the width of the hook curve, β(t), between their start and end points, and the height, α(t), in the limit of vanishing saturation.

The mean expression index characterizes the mean expression level of present probes of the chip,(24)

<..>_R > 1 _denotes averaging over all probe-sets with a S/N-ratio R(0) > 1.

The limiting values of the "start" and "end" coordinates of the hook curve refer to vanishing (R→0) and infinite (R→∞) values of the S/N-ratio, respectively:(25)

and(26)

The approximation for the start-coordinates neglects saturation of non-specific binding.

The time dependence of the hook-parameters simply considers the fact that washing shifts and deforms the hook curve in both the vertical and horizontal directions (see Figure 17 for illustration). For example, the end point moves upwards by Δ(∞,t) after washing owing to the stronger removal of specific transcripts from the MM compared with the PM (w^PM,S^(t)> w^MM,S^(t)). Note also that the parameter Δ(∞,t) defines the limiting ratio of the PM and MM probes upon saturation (R→∞) as a function of time. Before washing it vanishes (Δ(∞,0) = 0) because PM and MM probes fully saturate at the same intensity level. Δ(∞,t) increases upon washing because the MM probes are more strongly washed-off than the PM-probes, giving rise to different observed saturation levels.

The incremental width of the curve can be decomposed into two contributions due to washing of non-specific (h = N) and specific (h = S) transcripts from the PM and MM probes,(27)

The contributions δβ^N^(t) and δβ^S^(t), shift the increasing and decreasing branch of the hook curve towards smaller abscissa values, respectively (see Figure 17). Analogously one gets for the increment of the height of the hook(28)

Comparison with Eq. (25) provides for the specific and nonspecific contributions δα^s^(t) = Δ(∞, t) and δα^N ^(t) ≈ 0, respectively. Hence, increases of the height of the hook curve with washing reflects essentially the diverging limiting washing levels of the PM and MM.

### Re-parametrization of the standard hook analysis

The standard hook method doesn't explicitly consider probe-specific washing in its original version (i.e. apply Eqs. (20) - (26) with w^P,h^(t) = 1) [10]. Adsorption is predicted to follow the hyperbolic function (Eq. (2)) with the same argument, namely the binding strength, in the numerator and denominator as well. Comparison of Eqs. (2) and (7) reveals that washing affects only the numerator by down-weighting the binding strength. Let us define the modified probe occupancy of the hyperbolic form(29)

It formally applies the washing functions also in the denominator in contrast with Eq. (7). Eq. (29) underestimates the denominator compared with Eq. (7) because of w^P,N^(t > 0) < 1. However it formally obeys the hyperbolic form of the adsorption isotherm. The denominator of the adsorption law describes the saturation behaviour which is relevant at large binding strengths, e.g. at large transcript concentrations and/or binding constants. In expression analysis it acts basically as a correction term which only slightly modifies the intensity response at small and intermediate transcript concentrations. Hence, Eq. (29) slightly attenuates the saturation behaviour compared with that predicted by the correct equation (7) however with relative low impact with respect to the shape of the hook curve. On the other hand, the practical impact is high because it allows using the standard hook analysis algorithm (and available program tools). Particularly, the modified binding isotherm Eq. (29) provides the 'standard' equations for the hook coordinates(30)

This equation is used in our standard microarray analyses without explicit consideration of the washing functions. Note that the only difference between Eqs. (30) and (20) is the argument of the saturation term which is either set to its initial value (t = 0) in Eq. (20) or to the running washing time t > 0 in Eq. (30). Figure 17 illustrates emprically that Eq. (30) (dashed curve) accurately reproduces the washing hook Eq. (20) in the range of accessible experimental data if one uses the modified width-parameter(31)

It simply defines the end-point of the hook-graph in such a way that it intersects the abscissa in the middle between the Σ-coordinates of the hooks before (t = 0) and after washing (t > 0), i.e.,.(32)

In summary, the 'standard' hook analysis based on the Langmuir adsorption isotherm can also be applied, to a good approximation, in the more realistic case of post-hybridization washing. The stoichiometric characteristics derived from the 'hook' parameters should be re-interpreted from stoichiometries after hybridization (see ref. [10] for details) into stoichiometries after washing (see Eqs. (21) - (26)): For example, the start coordinates (Eq. (22)) and the PM/MM-gain α (Eq. (23)) refer to the respective probe populations which survived after washing. The obtained width parameter β underestimates the washing effect of specific transcripts by 50% (Eq. (31)), however with small impact because the effect of washing on non-specific transcripts largely exceeds that on specific ones.

### Kinetic analysis of the hook parameters

Let us describe the long-time kinetics of the washing functions (see Figure 10) by the same exponential time-dependence used in Eq. (10), i.e.(33)

Eq. (33) is equivalent to a washing function w^P,h^(t) = exp(-k^P,h^(t)·t) with a slowly varying washing rate k^P,h^(t) which decays hyperbolically with the number of washing cycles, i.e.(34)

Insertion of Eq. (33) into the equations for the hook-parameters (Eqs. (21)- (26)) then provides the set of linear equations which express the kinetic exponents of the hook parameters as a combination of the kinetic exponents of the washing functions:(35)

After rearrangement one gets expressions for the specific and non-specific washing exponents of the PM and MM,(36)

which characterize the mean long-time kinetics of washing functions of the respective probes.

### Hook analysis of the asymptotic washing level

The PM/MM log-difference was calculated for the asymptotic washing level(37)

(see Figure 7). The respective theoretical values can be approximated if one writes the asymptotic washing level of the P = PM and MM probes as the weighted average of the respective contributions due to specific and non-specific binding,(38)

where the W _∞ _(K ^P, h^) are given by Eq. (18). For the weighting factor we use the function, , where x^P,S ^denotes the fraction of specific hybridization and γ_f_≈1.5 is an adjustable exponent. The S/N-ratio of the PM-probes, R^PM^, is defined in Eq. (21). The respective S/N-ratio of the MM-probes and their specific binding constant is reduced by the factor 10^-α(0)^, i.e. K^MM,S ^= K^PM,S^·10^-α(0) ^< 1 and R^MM ^= R^PM^·10^-α(0) ^where the exponent is defined in Eq. (23).

Part b of Figure 17 compares the hook-plot of the log-intensities (Δ-vs-Σ, Eq. (20) with t = 0) with the hook-plot of the asymptotic washing level which was calculated after insertion of Eq. (38) into Eq. (37) (Δ_w_-vs-Σ). Note that the maximum height of the former curve is directly related to α(0), i.e. to the log difference of the specific binding constants of the PM and MM which is governed by the single-mismatch design of the latter probes (Eq. (23)). Contrarily, the maximum of the w∞-hook is inversely related to the binding constant for non-specific binding,  where we assume K^PM,N ^≈ K^MM,N ^= K^N ^<< K^PM,S^. For the asymptotic level of Δ_w _one gets(39)

where the approximation on the right side assumes γ_w_·α(0)>1, or equivalently K ^MM, S ^< K ^PM, S^.

### The effect of washing on the positional dependent nucleotide sensitivities

Each probe intensity can be decomposed into the mean 'set-contribution' and into a probe- and thus sequence-specific incremental term,(40)

where each probe set interrogates the abundance of one transcript.

The hook method fits the intensity increment using the positional-dependent sensitivity model to correct the raw probe intensities for sequence specific effects of [10,26,33]. The model approximates the increments of the intensity by the sum(41)

with the constraints ⟨δ log I⟩_p-set _= 0 and . B_pk _denotes the nucleotide-letter at sequence position k of probe p. Here we consider a single-base model for the sensitivities σ_k_^P,h ^for sake of simplicity. In general, the hook method uses a nearest neighbour or even next-nearest neighbour sensitivity model as standard to correct the intensities for sequence effects [10].

With Eqs. (7), (5) and (6) one gets after differentiation for the special cases of predominant specific (h = S) and non-specific (h = N) hybridization at constant transcripts concentration ([h] = const)(42)

where the last and the middle term in the brackets account for saturation and washing, respectively. In analogy with Eq. (40) we decompose the binding constant into the sequence-independent mean value over all probes and into the sequence-dependent incremental term, . In the linear range far from saturation (K_0_^P,h^[h]< < 1) and in the range of saturation (K_0_^P,h^[h]→∞) Eq. (42) simplifies into(43)

The leading term in Eq. (42) is given by the logarithm of the binding constant. It is directly related to the free energy of binding (see also the text above Eq. (6)) which in turn can be modelled by a sum of positional dependent base-specific terms,(44)

After comparison of Eqs. (42), (44) and making use of the special cases in Eq. (43) one gets the positional sensitivity terms,(45)

with δ_sat _= 0 in the linear range and δ_sat _= -1 in the saturation range. For the incremental contribution due to washing one obtains(46)

According to Eqs. (45) and (46), the absolute values of the sensitivities are expected to increase with washing time in agreement with the experimental results (see Figure 14).

## Authors' contributions

All authors carried out the study, read and approved the final manuscript.

## Supplementary Material

Additional file 1 **Additional file **1**: presents the Hook analysis of the washing experiment of Skvortsov et al., selected results of a pre-experiment using GeneChip Test3 arrays and contains a discussion of the design of the main experiment**. Click here for file
