# Supplementary material for: Washing scaling of GeneChip microarray expression
Source: BMC Bioinformatics. 2010 May 28;11:291. doi: 10.1186/1471-2105-11-291 (PMC2901370; doi:10.1186/1471-2105-11-291)
Supplement: Additional file 1 — presents the Hook analysis of the washing experiment of Skvortsov et al., selected results of a pre-experiment using GeneChip Test3 arrays and contains a discussion of the design of the main experiment. [file 1471-2105-11-291-S1.PDF]

Supporting Information

Washing scaling of microarray expression

Hans Binder[1*], Knut Krohn[2] and Conrad J. Burden[3]

[1] Interdisciplinary Centre for Bioinformatics of Leipzig University, D-4107 Leipzig, Haertelstr. 16-18
[2] Interdisciplinary Center for Clinical Research, Medical Faculty; University of Leipzig, D-04107 Leipzig, Inselstr. 22
[3] Mathematical Sciences Institute, Australian National University, Canberra, A.C.T.0200, Australia

* Corresponding author: E-mail: binder@izbi.uni-leipzig.de, fax: ++49-341-9716679

1. Hook analysis of the washing experiment of Skvortsov et al.

To illustrate the benefits of the hook approach we transform the intensity-data of a second washing experiment published recently by Skvortzov et al. [1] into hook-graphs (see Supplementary Figure 1). In this particular experiment the authors labelled only a small number of 18-20 transcripts each of which is interrogated by one 'spiked probe set' to filter out the effect of specific hybridization. With this limited number of labelled transcripts the smoothing algorithm of the standard hook method partly fails because it takes running averages over the intensities of about one hundred probe sets, a number that largely exceeds the number of labelled transcripts. As a consequence, the obtained hook curves lack the S-, sat- and as-ranges because of the small number of strongly expressed probe sets (see part a of Supplementary Figure 1 and Figure 7 for the definition of the hybridization ranges). In part b of Supplementary Figure 1 we therefore plot the individual probe-set level data without smoothing. The obtained data clouds are well described by theoretical hook curves. The spiked probe sets (solid symbols) essentially accumulate in the sat-range due to their relatively strong specific hybridization with the spikes. Contrarily, the probe sets not interrogating the targets (so-called 'empty' probe sets) are mostly found in the N-hybridization range owing to cross hybridization with the spikes (see the open symbols). The smoothed hook curves essentially reflect the behaviour of the N-range which is most strongly affected by washing.

The 'washing-cycle experiment' of Skvortsov et al. uses a modified fluidic script similar to that applied to chip A in our study: Particularly, it scans the array once before and two-times after stringent washes (see ref. [1] for details). The respective hook data undergo virtually the same changes as in our study (compare part a and b of Supplementary Figure 1 with Figure 8). Namely, the N- range shifts markedly towards smaller values paralleled by the increase of the height of the hook curve. Repeated washing is much less effective than the first washing step. Note that the results also agree quantitatively with ours: the observed shift of the N-range, $\delta\beta^N$~0.8, agrees in both experiments despite the different chip types used by us (Human HG-U133plus2 array) and Skvortsov et al. (Drosophila DG-1 array).

In a second 'hybridization time experiment' Skvortsov et al. hybridized two microarrays differently before washing, one for 16 hours and one for 40 hours. The hook-analysis clearly reveals that longer hybridization markedly decreases the horizontal shift of the hook curves before and after washes. Hence, longer hybridization clearly decreases the efficiency of washing. This trend has been explained by the better equilibration which on the average stabilizes probe/target duplexes [1]. The hook-data show that the specifically hybridized spike-probe sets in the S- and as-ranges of the hook curve are much less affected by the hybridization time than non-specifically hybridized empty probes in the N-range (see part d of Supplementary Figure 1). In other words the resistance of non-specific hybridization to washing gains more strongly with hybridization time on a relative scale than specific hybridization. In consequence, longer hybridization times give rise to larger non-specific signals, which effectively reduces the effect of washing and this way deteriorates the signal-to-noise ratio of the expression measures.

These examples demonstrate that the hook-presentation of microarray data allows the simple and straightforward characterization of the effect of washes on the degree of probe/target binding. Note

that our approach does not require selective labeling of the spikes to differentiate non-specific from specific hybridization.

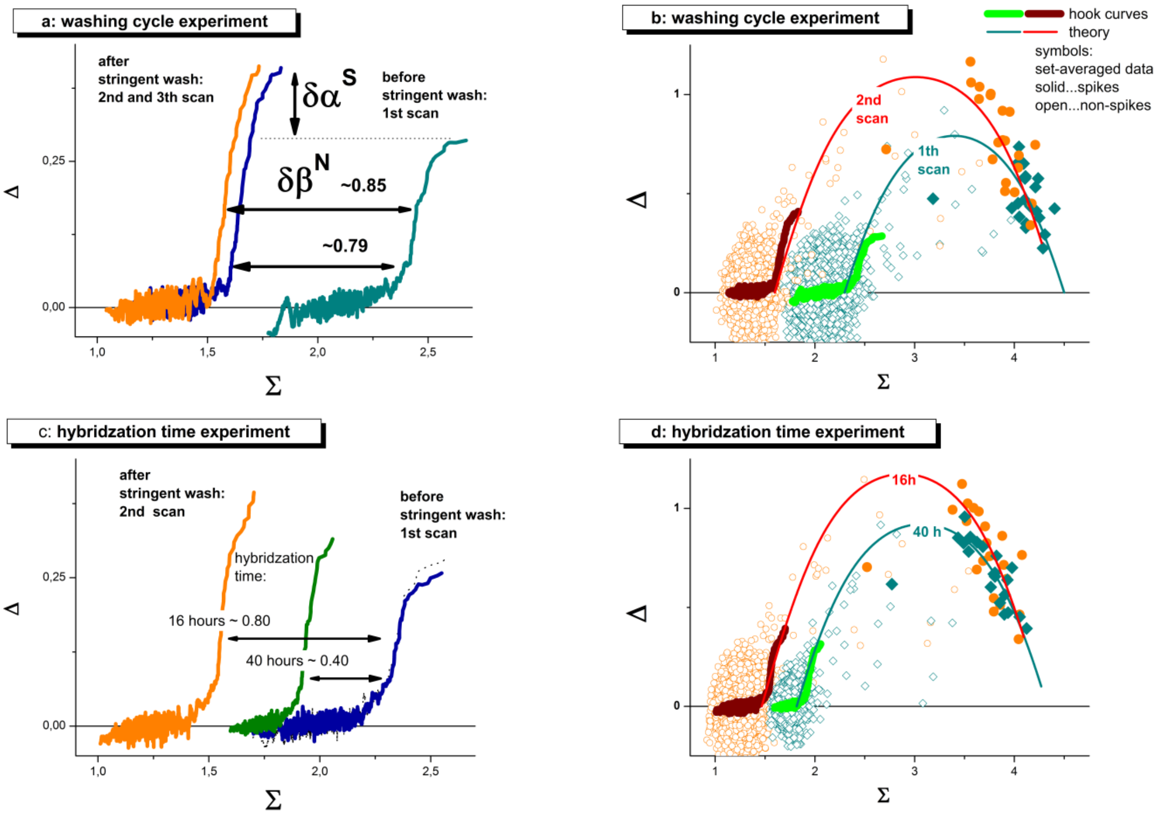

Supplementary Figure 1: Hook-analysis of the washing experiments of Skvortsov et al. [1]. In the washing cycle experiment hybridized arrays are scanned before and after stringent washing similar to array A in our experiment (see Figure 1). In contrast to our study only selected spikes referring to 20 probe sets are fluorescently labeled. The hook curves therefore show essentially only the N- and the mix-ranges due to the small number of data points in the S- and as-ranges (panel a, see also Figure 7 and panel b). The horizontal shift between N-ranges ($\delta\beta^N$) of the hook curves characterizes the mean washing effect of non-specifically hybridized probes. Panel b shows the probe set averaged values of the spikes (solid symbols) and of the empty probes (non-spikes, open symbols) and also theoretical curves calculated using Eq. (20). The empty probes are cross hybridized by the spikes. Panel c shows that longer hybridization times (40-versus-16 hours) reduce the shift of the hook curve. The better stabilization of probe-bound targets obviously decreases the effect of washing. Panel d shows probe-set level data of the hybridization time experiment after washing (see panel b for assignments). The strongly bound spikes are only weakly affected by the washing time.

2. Washing pre-experiment using GeneChip Test3 arrays and the design of the main experiment

We performed a pre-experiment to test the fluidic script applied in the washing-scanning protocol prior to the main experiment described in the paper. In the pre-experiment two Test3 GeneChip arrays are used which were specially designed for testing purposes and quality control [2]. These arrays contain a strongly reduced number of probes (about 6,000) referring to different organisms. In our pre-experiment we used two chips hybridized with different RNA-preparations which were extracted from rat hypothalamus (array P1) and small intestine (array P2). Washing-scanning cycles were performed with both arrays according to the protocol applied to array A in the first series of the main experiment prior to re-labeling, except that only five stringent washes were used in the last cycle instead of ten washes (see Fig. 1).

Supplementary Figure 2 reveals similar washing kinetics of the logged non-specific background intensity for the two arrays used in of the pre experiment and for array A later studied in the main experiment (see also Figure 10). In both experiments, the first two stringent washes applied after the first scanning round decrease the logged intensity roughly by the same increment as the subsequent ten-fifteen washes applied after the second-third scans. The absolute effect of washing is slightly smaller for the Test3 arrays for unknown reasons but virtually identical for the two arrays studied in the pre-experiment. Note that the background level of the Test3 arrays markedly exceeds that of the HGU133plus2 array possibly because this array type contains a relatively large fraction of probes taken from different organisms which mismatch the target RNA to a large amount.

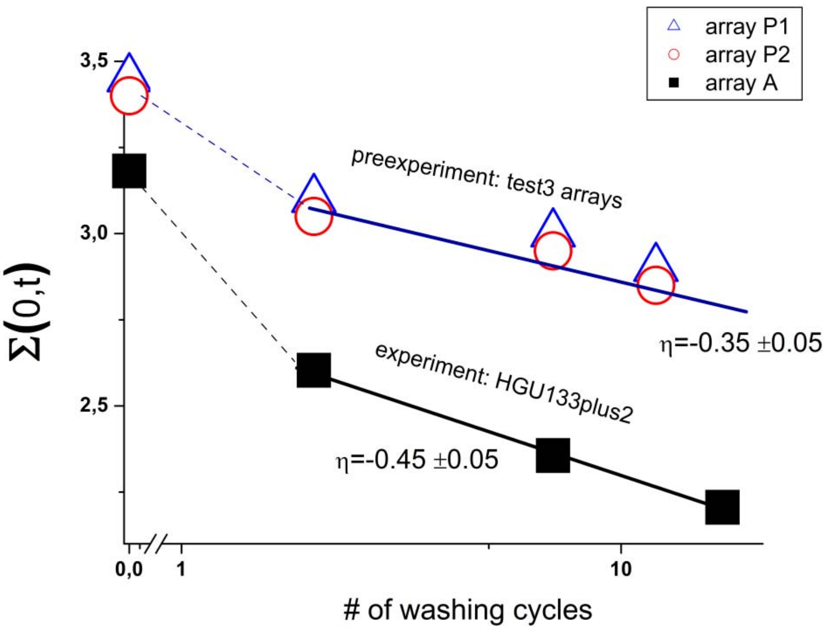

Supplementary Figure 2: Washing kinetics of the mean logged intensity due to non-specific hybridization of array A (solid squares) and the arrays of the pre-experiment as a function of the number of washing cycles. The data of array A are re-plotted from Fig. 10. η is the slope of the linear approximation of the decay.

Also individual probe intensities selected from the Test3 arrays in the range of small, intermediate and large intensities show very similar kinetics to the probes taken from the HGU133plus2 arrays (data not shown, see also Fig. 4a). We analysed all probe intensities of the test3 arrays using the empirical decay law given by Eq. 8. Supplementary Figure 3 plots the limiting values of the washing function (Eq. 8 and 9) as a function of the probe intensity. The data sets obtained from the main and the pre-experiment show essentially the same properties, namely a strong washing effect for low intensity probes below a certain intensity threshold. Above this threshold the probes progressively resist against washing in both experiments. Both data sets can be described by the same form of the washing function, but with slightly varied parameters to account for the shifted threshold in the pre-experiment (see the legend of Supplementary Figure 3). This shift corresponds to the higher level of the non-

specific background intensity in the pre-experiment. The critical exponent defining the slope of the washing function is identical for both experiments.
The results of the pre-experiment agree also in other respects with that of the main experiment (data not shown): For example, the washing efficiency doesn't depend on the probe type (PM or MM) for identical probe intensities, but it largely differs for each PM/MM-pair in the range of specific binding where the intensity of the PM typically exceeds that of the MM.
In summary, the pre-experiment provides virtually identical results for the basic washing characteristics for the two studied Test3 arrays which differ only with respect to the sample RNA. They are technical replicates which are treated identically after hybridization. Comparison with the results of the main experiment, and particularly with array A, also provides good agreement with respect to all studied longitudinal washing characteristics. Larger differences of the mean intensity levels of selected probe ensembles are due to the specifics of the array types used and are virtually not or are only weakly related to washing. In previous publications we studied the amplitudes and origin of chip-to-chip and also of chip type-to-chip type variability of the used hook parameters in detail [3-5].

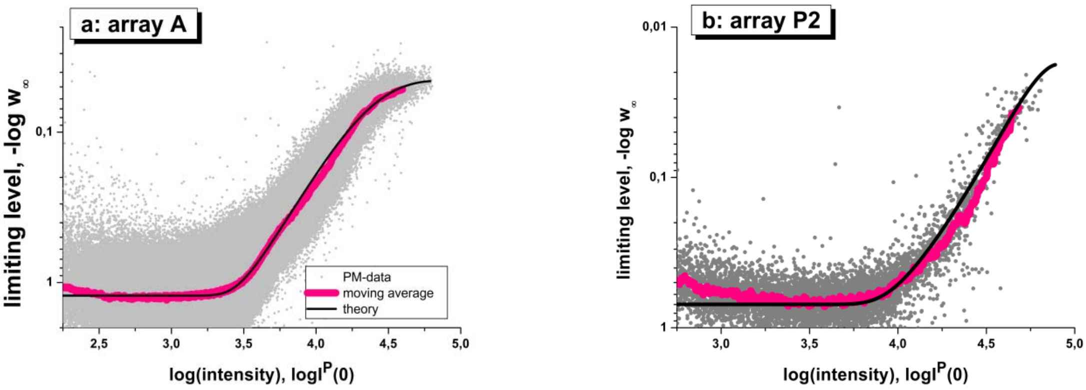

Supplementary Figure 3: Limiting values of the washing function for all PM probes of array A (panel a) and array P2 (panel b) as a function of the initial probe intensity $\log I^{PM}(0)$. Probe level data are shown by dots (Eq. 8 and 9). The theoretical curve was calculated using Eq. 17. Part a re-plots the data from Figs. 5a (data) and 15c (theory) where the theoretical curve was calculated with w_{max}=0.9, w_{min}=0.06, γ=1.6, logM=4.8 and a'=0.1. The theoretical curve in part b was calculated with w_{max}=0.96, w_{min}=0.2, γ=1.6, logM=4.9 and a'=0.22. Note that the number of individual probe intensities is smaller by two orders of magnitude for the test3 array ($O(10^3)$ versus $O(10^5)$).

The pre-experiment has shown that technical replication gives rise to relatively low variance of the array characteristics studied. The tiny effect of biological variation confirms the trivial fact that washing is driven mainly by physico-chemical factors. Subtle expression differences between the different RNA extracts are obviously averaged out and thus of minor relevance for our study. The treatment of the three arrays used in the main experiment was therefore designed as a time series using identical RNA material and identical treatment protocols, the only difference being the number of stringent washes between the scans. This scanning of the three arrays at different times allows the efficient use of the microarrays because it gives a denser spread of time points than using the same time points for all three arrays. Moreover, it allows one of the arrays to be treated by the standard Affymetrix washing protocol and establishes whether the results fit into the time dependence determined by the other arrays.
Cross array comparisons include variance effects due to the different fluidic scripts, independent hybridizations and variance associated with technical replication. The errors obtained are consequently maximum estimates which exceed the expected errors estimated from simple replicates which only include variance effects due to independent hybridizations and technical replication. The estimated regression error of 10-25% for the slopes of array A and B shown in Fig. 10 of the paper should be therefore judged as maximum error estimates. For simple replication we indeed obtained much better agreement for arrays P1 and P2 (see Supplementary Figure 2). The pooling errors due to the scattering of probe-level data about the mean washing function were considered and illustrated as ± 50%

deviations in Fig. 15. The discovery of the origin of these deviations and the search for appropriate probe-level correction goes beyond the scope of the present paper.

It is also important to recall that our washing study doesn't focus on single-gene properties but instead mainly uses summary characteristics averaged over large probe collectives collected from each array. This approach is justified by the physico-chemical background of washing which attributes the effect under study to the free energy of probe/target binding or to the related probe intensity. Here we have typically hundreds-to-thousands of individual probe sequences which can be pooled per intensity value (see Figs. 5 and 15 and the associated data). Moreover, our results are mainly based on relative changes as a function of the washing time via longitudinal sampling for each array at different time points (see Figs. 10 and 12). This approach reduces chip specific factors which affect, for example, the average intensity level but does virtually not affect how the intensity changes with washing time. Taken together, our analysis of washing characteristics is mainly based on relative changes of pooled data with much better resolution than typical expression analyses comparing single-gene specific data between different samples.

Such longitudinal sampling in time-series experiments [6-8] and pooling methods [9-11] constitute accepted strategies in microarray studies for estimating differential expression 'without replicates'. Pooling strategies which group genes of similar expression values have been also proven to handle small sample sizes in terms of accurate error estimates [12]. Storey et al. [13] apply functional data analysis to microarray time series experiments to identify differentially expressed genes sampled in longitudinal direction (each individual is sampled at more than one time point; different individuals must not be sampled at identical time points). Even though we do not follow precisely any of the methods used in these publications, we do follow the same basic strategy based on functional data analysis and pooling.

3. References

1. Skvortsov D, Abdueva D, Curtis C, Schaub B, Tavare S: **Explaining differences in saturation levels for Affymetrix GeneChip(R) arrays**. *Nucl Acids Res* 2007, **35**(12):4154-4163.
2. Affymetrix: **GeneChip Test3 array**. *Technical Note* 2001.
3. Binder H, Preibisch S: **"Hook" calibration of GeneChip-microarrays: Theory and algorithm**. *Algorithms for Molecular Biology* 2008, **3:12**.
4. Binder H, Preibisch S, Berger H: **Calibration of microarray gene-expression data**. In: *Methods in Molecular Biology*. Edited by Grützmann R, Pilarski C, vol. 375-407. New York: Humana Press; 2009.
5. Binder H, Bruecker J, Burden CJ: **Non-specific hybridization scaling of microarray expression estimates - a physico-chemical approach for chip-to-chip normalization**. *J Phys Chem B* 2009, **113**:2874-2895.
6. DeCook R, Nettleton D, Foster C, Wurtele ES: **Identifying differentially expressed genes in unreplicated multiple-treatment microarray timecourse experiments**. *Computational Statistics & Data Analysis* 2006, **50**(2):518-532.
7. Han X, Sung WK, Feng L: **Identifying differentially expressed genes in time-course microarray experiment without replicate**. *J Bioinform Comput Biol* 2007, **5**:281-296.
8. Billups S, Neville M, Rudolph M, Porter W, Schedin P: **Identifying significant temporal variation in time course microarray data without replicates**. *BMC Bioinformatics* 2009, **10**(1):96.
9. Hein A-MK, Richardson S, Causton HC, Ambler GK, Green PJ: **BGX: a fully Bayesian integrated approach to the analysis of Affymetrix GeneChip data**. *Biostat* 2005, **6**(3):349-373.
10. Hein A-M, Richardson S: **A powerful method for detecting differentially expressed genes from GeneChip arrays that does not require replicates**. *BMC Bioinformatics* 2006, **7**(1):353.
11. SiliconGenetics: **Cross-Gene Error Model**. *Technical Note* 2002, http://www.silicongenetics.com/Support/GeneSpring/GSnotes/analysis_guides/error_model.pdf.
12. Jain N, Thatte J, Braciale T, Ley K, O'Connell M, Lee JK: **Local-pooled-error test for identifying differentially expressed genes with a small number of replicated microarrays**. *Bioinformatics* 2003, **19**(15):1945-1951.
13. Storey JD, Xiao W, Leek JT, Tompkins RG, Davis RW: **Significance analysis of time course microarray experiments**. *Proceedings Of The National Academy Of Sciences Of The United States Of America* 2005, **102**(36):12837-12842.
